# Sphingomyelin regulates astrocyte activity by regulating NF-κB signaling via HDAC1/3 expression

**DOI:** 10.1016/j.jlr.2025.100933

**Published:** 2025-11-04

**Authors:** Ryo Kadowaki, Hana Hirose, Gai Takimoto, Takafumi Kohama, Hiroyuki Nakamura

**Affiliations:** Laboratory of Chemical Pharmacology, Graduate School of Pharmaceutical Sciences, Chiba University, Chuo-ku, Chiba, Japan

**Keywords:** abnormal astrocyte activation, sphingomyelin, NF-κB signaling, histone deacetylase, p65 acetylation, neurodegenerative diseases

## Abstract

Astrocytes comprise approximately 40% of CNS cells and have pivotal roles in brain functions. Under steady-state conditions, astrocytes maintain homeostasis in the CNS through the uptake or release of neurotransmitters. However, in neurodegenerative conditions, astrocytes are activated by inflammatory cytokines, such as interleukin-1alpha (IL-1α) and TNF-α, which are released from activated microglia. Activated astrocytes release several inflammatory cytokines and neurotoxic substances, resulting in neuronal injury. Sphingolipids are a series of bioactive lipids involved in several biological processes, such as apoptosis, inflammatory response, cell cycle, and immune response. SM is a sphingolipid that is a major component of the cellular membrane and is also involved in inflammatory responses. We report that SM promotes IL-1α/TNF-α-induced expressions of representative astrocyte mRNAs and astrocyte activation through the NF-κB pathway. In contrast, reduction of SM by knockdown of sphingomyelin synthase 1 (SMS1) and/or SMS2 suppresses astrocyte activation. Furthermore, removal of SM by the blockade of ceramide transfer protein suppresses astrocyte activation via the induction of histone deacetylase (HDAC) 1 and HDAC3; subsequently, the levels of acetylated p65 (Lys 310) are reduced, leading to the suppression of the NF-κB pathway. Our findings further the understanding of the regulation of astrocyte activation by sphingolipids.

Astrocytes are glial cells that maintain the CNS by modulating levels of extracellular neurotransmitters, including glutamate, brain-derived neurotrophic factor, and monoamines (1, 2). Conversely, astrocytes are abnormally activated by inflammatory cytokines, such as interleukin-1α (IL-1α) and TNF-α, which are released from activated microglia in neurodegenerative diseases that include Alzheimer’s disease and multiple sclerosis (3, 4). Activated astrocytes release neurotoxic substances, including reactive oxygen species and inflammatory cytokines, resulting in neuronal death (3, 5). Moreover, activated astrocytes damage neurons by downregulating glutamate transporters and disrupting CNS homeostasis, consequently exacerbating neurodegenerative diseases (6). Modulating the activation of astrocytes induced by inflammatory cytokines could be a novel therapeutic target for neurodegenerative diseases.

Sphingolipids are lipids with a long-chain amino alcohol backbone. These lipids participate in various physiological functions, such as cell survival, migration, and apoptosis (7, 8). SM is the most abundant sphingolipid in mammalian cells and is mainly localized in the plasma membrane and other cellular membranes (9, 10). SM acts as a structural molecule in membranes and is also involved in inflammatory responses by forming an interaction with ceramide, called the SM cycle (11, 12).

The lactosylceramide glycosphingolipid regulates astrocyte activation via the cytosolic phospholipase A2-mitochondrial antiviral signaling protein-NF-κB axis (13). Similarly, inhibition of the sphingosine-1-phosphate receptor, a G protein-coupled receptor that mediates sphingosine-1-phosphate signaling, suppresses astrocyte activation and ameliorates chronic progressive inflammation in an experimental mouse model of autoimmune encephalomyelitis (14). These results imply that sphingolipids can control astrocyte activation. However, the details of the involvement of sphingolipids in astrocyte activity remain unknown.

We investigated the effects of SM on IL-1α/TNF-α-induced astrocyte activation, which mimics astrocyte phenotypes in neurodegenerative disease conditions, using HASTR/ci35 cells, a human astrocyte-like cells. Increasing intracellular SM levels by inhibiting SM degeneration accelerated the activation of astrocytes induced by IL-1α/TNF-α. By contrast, reducing intracellular SM levels by blocking SM metabolic enzymes suppressed this induced activation by attenuating the NF-κB signaling pathway. Our findings provide novel insights into sphingolipid-mediated astrocyte activation.

## Materials and Methods

### Antibodies

Primary antibodies to cyclooxygenase-2 (COX-2; Cat# 12282), acetyl-Lys^310^-p65 (Cat# 12629S), NF-κB inhibitor alpha (IκBα; Cat# 4814S), and phospho-Ser^32^-IκBα (Cat# 2859) were obtained from Cell Signaling Technology. Antibody to GAPDH (Cat# 016-25523) was purchased from WAKO. Antibody to ceramide transport protein (CERT; Cat# ab72536) was purchased from Abcam. Antibody to FLAG-M2 antibody (Cat# F1804) was purchased from Merck. Antibodies to p65 antibody (Cat# sc-8008), phospho-Ser^536^-p65 (Cat# sc-136548), TNF receptor 1 (TNFR1; Cat#sc-9436), histone deacetylase 1 (HDAC1; Cat# sc-81598), HDAC3 (Cat# sc-376957), calnexin (Cat# sc-46669), lamin B1 (Cat# sc-374015), and flotillin-1 (Cat# 74566) were obtained from Santa Cruz Biotechnology.

The secondary antibodies used in this study were horseradish peroxidase-linked anti-rabbit IgG (Cat# 7074) and anti-mouse IgG (Cat# 7076) purchased from Cell Signaling Technology. StarBright Blue 700 goat anti-mouse IgG (Cat# 12004158) was obtained from Bio-Rad. Goat anti-mouse IgG (H + L) Cross-Adsorbed secondary antibody, Alexa Fluor™ 488 (Cat# A-11001) and Goat anti-rabbit IgG (H + L) Cross-Adsorbed Secondary antibody, Alexa Fluor™ 488 (Cat# A-11008) were obtained from Invitrogen.

### Reagents and plasmid

Recombinant human IL-1α (Cat# 200-01A) and TNF-α (Cat# 300-01A) were purchased from PeproTech. GW4869 (hydrochloride hydrate; Cat# 13127), bovine spinal cord sphingomyelin (Cat# 22674), BAY 11-7082 (Cat# 10010266), T-5224 (Cat# 22904), SM (d18:1/C17:0) (Cat# 24354), SM (d18:1/C20:0) (Cat# 24450), SM (d18:1/C22:0) (Cat# 24451), SM (d18:1/C26:0) (Cat# 9003457), ceramide (d18:1/C6:0) (Cat# 62510), ceramide (d18:1/C14:0) (Cat# 22531), ceramide (d18:1/C16:0) (Cat# 10681), ceramide (d18:1/C17:0) (Cat# 22532), ceramide (d18:1/C18:0) (Cat# 19556), ceramide (d18:1/C18:1) (Cat# 24397), ceramide (d18:1/C20:0) (Cat# 10724), ceramide (d18:1/C22:0) (Cat# 22533), ceramide (d18:1/C24:1) (Cat# 62530), and ceramide (d18:1/C24:0) (Cat# 62535) were purchased from Cayman Chemical. SM (d18:1/C12:0) (Cat# LM2312), SM (d18:1/C14:0) (Cat# 860688P), SM (d18:1/C16:0) (Cat# 860584), SM (d18:1/C18:0) (Cat# 860586P), SM (d18:1/C18:1) (Cat# 860587P-5), SM (d18:1/C24:1) (Cat# 860593P), and SM (d18:1/C24:0) (Cat# 860592P) were purchased from Avanti Polar Lipids. Ceramide (d18:1/C26:0) (Cat# FC19613) was obtained from Biosynth Carbosynth. SR 11302 (Cat# HY-15870) and TPCA-1 (Cat# HY-10074) were obtained from MedChem Express. Lysenin and lysenin antisera (Cat# 4802-v and 14802-v, respectively) were purchased from Peptide Institute Inc. (Osaka, Japan). Varproic acid sodium (Cat# S0894) was obtained from TCI (Tokyo, Japan). pcDNA3.1+/C-(K)DYK-p65 (Cat# OHu26911) was obtained from GenScript.

### Culture and differentiation of human immortalized astrocytes

HASTR/ci35 human immortalized astrocytes provided by Dr Furihata (School of Pharmacy, Tokyo University of Pharmacy and Life Sciences) were routinely cultured at 33°C in an atmosphere of 5% CO_2_/95% air in DMEM (Nacalai) containing 1% N2 Supplement (WAKO), 2 mM L-Alanyl-L-Glutamine (WAKO), 10% FBS, 1% penicillin-streptomycin and 4 μg/ml blasticidin S (Kaken Pharmaceutical). This medium is hereafter referred to as complete astrocytes medium (CAM). HASTR/ci35 cells were cultured in type I collagen-coated dishes (WAKO). HASTR/ci35 cell differentiation was performed as previously described (15). Briefly, cells were rinsed twice with PBS and then cultured in FBS-free CAM (hereafter referred to as serum-free astrocyte medium, SFAM) at 37°C in an atmosphere of 5% CO_2_/95% air.

### Western blot analysis

The cells were washed twice with ice-cold PBS and collected in Laemmli buffer. The cells were lysed by sonicating using a probe sonicator. Protein was quantified using a BCA protein assay kit (Nacalai), and 2-mercaptoethanol (2-Me; WAKO) was added to cleave disulfide bonds. After boiling at 100°C for 5 min, the proteins were resolved by SDS-PAGE and transferred to a PVDF membrane. The membrane was blocked with 5% BSA/TBS-Tween (TBS-T) for 1 h at room temperature and then incubated with primary antibodies (1:1000) overnight at 4°C. After washing three times with TBS-T, the membrane was incubated with secondary antibodies (1:3000-1:5000) for 1 h at room temperature. Protein bands were detected using a ChemiDoc MP imaging system (Bio-Rad). The obtained images were analyzed using Image Lab Software (Bio-Rad).

### Treatment with small-interfering RNA (siRNA)

Cells were washed twice with serum-free DMEM, transfected with siRNAs using Lipofectamine RNAiMAX (Invitrogen) following the manufacturer’s instructions, and cultured in SFAM. After 48 h, the cells were washed twice with serum-free DMEM and treated with IL-1α (3 ng/ml) and TNF-α (50 ng/ml) for 24 h. The cells were collected for subsequent experiments. siRNA sequences used in this study are listed in Supplemental Table S1.

### Immunohistochemistry

Cultured cells were fixed with 4% paraformaldehyde phosphate buffer solution (PFA) (Cat# 09154-85; Nacalai) for 15 min at room temperature. The fixed cells were blocked and permeabilized by 3% BSA/0.1% Triton-X mixed buffer for 1 h. Following that, the cells were incubated in 1% BSA/0.1% Triton-X containing anti-p65 antibody (1:250) overnight at 4°C. After washing with PBS, the cells were incubated with 1% BSA/0.1% Triton-X containing Alexa Fluor-conjugated anti-mouse IgG (1:500) for 1 h at room temperature, protected from light. After washing with PBS three times, the cells were sealed with 50% glycerol. Fluorescence images were acquired using a model LSM780 confocal microscope (Carl Zeiss).

### Total RNA isolation and quantitative real-time PCR (qPCR)

Total RNA was extracted using ISOGENⅡ (NIPPON GENE) following the manufacturer’s instructions. cDNA was prepared with ReverTra Ace® qPCR RT Master Mix with gDNA Remover (TOYOBO, Osaka, Japan). THUNDERBIRD Next SYBR® qPCR Mix (TOYOBO) and CFX Duet Real-Time PCR System (Bio-Rad) were used for qPCR. Primer sequences used in this study are listed in Supplemental Table S2. Relative mRNA expression levels were calculated using ΔΔCq method. The qPCR protocol involved an initial melting step of 95°C for 1 min, followed by 40 cycles of 95°C for 10 s as the melting step, and annealing at 60°C for 15 s.

### Lipid extraction and endogenous SM quantification

The cells were washed three times with ice-cold PBS and scraped off the culture surface. Before lipid extraction, protein quantification was performed using a protein assay BCA kit (Cat# 06385-00; Nacalai), and then protein content was normalized. Total lipids were extracted using the method of Bligh and Dyer. The organic layer was then dried using a centrifugal evaporator (EYELA). Dried lipid samples were resolved in 10 μl of chloroform:methanol (1:1, v/v), applied to Silica Gel 60 TLC plates (Merck), and separated using chloroform:methanol:water (65:25:4, v/v/v). The imaging of separated lipids was carried out, followed by our previous reports (16, 17, 18). Briefly, the Silica Gel 60 TLC plates were dipped into the 47% sulfuric acid and immediately heated at 150°C on a hot plate. The TLC images were obtained by the ChemiDoc MP imaging system (Bio-Rad).

### Lysenin staining

SM labeling was performed as previously described (16). Briefly, cultured cells on a cover glass were washed with PBS twice and then fixed with 4% PFA for 15 min. After treatment with 0.5% saponin for 15 min at room temperature, the cells were blocked with 2% BSA in PBS for 1 h at room temperature. The blocked cells were then treated with 1 μg/ml lysenin in 0.2% BSA in PBS for 1 h at room temperature, washed with PBS twice for 5 min each time, incubated with anti-lysenin antiserum in 0.2% BSA in PBS (1:250) for 1 h at room temperature, and treated with Alexa Fluor-conjugated anti-rabbit IgG in 0.2% BSA in PBS (1:500) for 1 h at room temperature. After washing with PBS three times, the cells were sealed with 50% glycerol. Fluorescence images were obtained a model LSM780 confocal microscope (Zeiss). For quantitative analyses, the mean intensity of obtained images was calculated using Zen Blue Edition software (Zeiss).

### SM dissolution for cellular treatment

SM dissolution was performed according to our previous study (16, 18). Briefly, SM powder was dissolved in a 98:2 (v/v) EtOH/dodecane solution and sonicated using Bransonic® (Cat# CPX2800H-J; Yamato Scientific) until the solution became clear. The dissolved SM was stored at −20°C until use. For cell treatment, the SM solution was added to the culture medium at the indicated concentrations and vigorously mixed.

### Lipid preparation for LC-MS/MS analysis

Lipid extraction from HASTR/ci35 cells for LC-MS/MS-based quantitative lipidomic analysis was performed following the method described by Merrill *et al.* (19) with minor modifications. Briefly, cells cultured in 100-mm dishes were washed twice with ice-cold PBS and collected into 1.5 ml tubes. 3 μM of internal standards (IS1: d18:1/C17:0-ceramide and d18:1/C17:0-SM) were added to the collected cells. Total lipids were then extracted using the Bligh and Dyer method as described above. The organic phase was concentrated using a centrifugal evaporator, and the lipid pellet was dissolved in methanol containing another internal standard (IS2: 100 nM d18:1/C6:0-ceramide and 50 nM d18:1/C12:0-SM). IS1 was used to correct for extraction efficiency, whereas IS2 was used to compensate for run-to-run variations in MS sensitivity. After LC-MS/MS analysis, raw data were normalized to IS1and IS2 values and further adjusted to protein concentration.

### LC-MS/MS-based quantitative lipidomic analysis

LC-MS/MS-based quantitative lipidomic analysis was performed using liquid chromatography and electrospray ionization-tandem mass spectrometry (LC-ESI-MS/MS; QTRAP®4500, AB SCIEX). Ceramide and SM species were separated from the lipid extracts as described in our previous study (20). For quantification, source, gas, and compound parameters were set according to the previous reported protocol (20) (see parameters for ceramide quantification). Additional parameters, including *m/z*, decluttering potential (DP), collision energy (CE), and collision cell exit potential (CXP), are summarized in Supplemental Table S3 (for ceramide) and Supplemental Table S4 (for SM). Calibration curves for each ceramide and SM molecular species were generated from standard samples (1–300 nM), and MS signal intensities of each sample were obtained using Analyst software (AB SCIEX). Lipid quantification was then performed using the MS signal intensity and dose-dependent calibration curves.

### Plasma membrane isolation

Plasma membrane isolation was performed using the Minute Plasma Membrane Protein Isolation Kit (Cat#SM-005; Invent Biotechnologies) according to the manufacturer’s instructions with minor modifications. The isolated membrane fraction was then diluted in PBS and used in subsequent experiments.

### Statistical analyses

All data are shown the mean ± standard error of the mean (SEM) for at least three independent experiments. An unpaired two-tailed Student’s *t* test was used to compare two groups. For more than two groups, data were analyzed using a one-way ANOVA followed by Tukey's test. *P* < 0.05 was considered statistically significant.

## Results

### SM promotes astrocyte activation

**Fig. 1:**
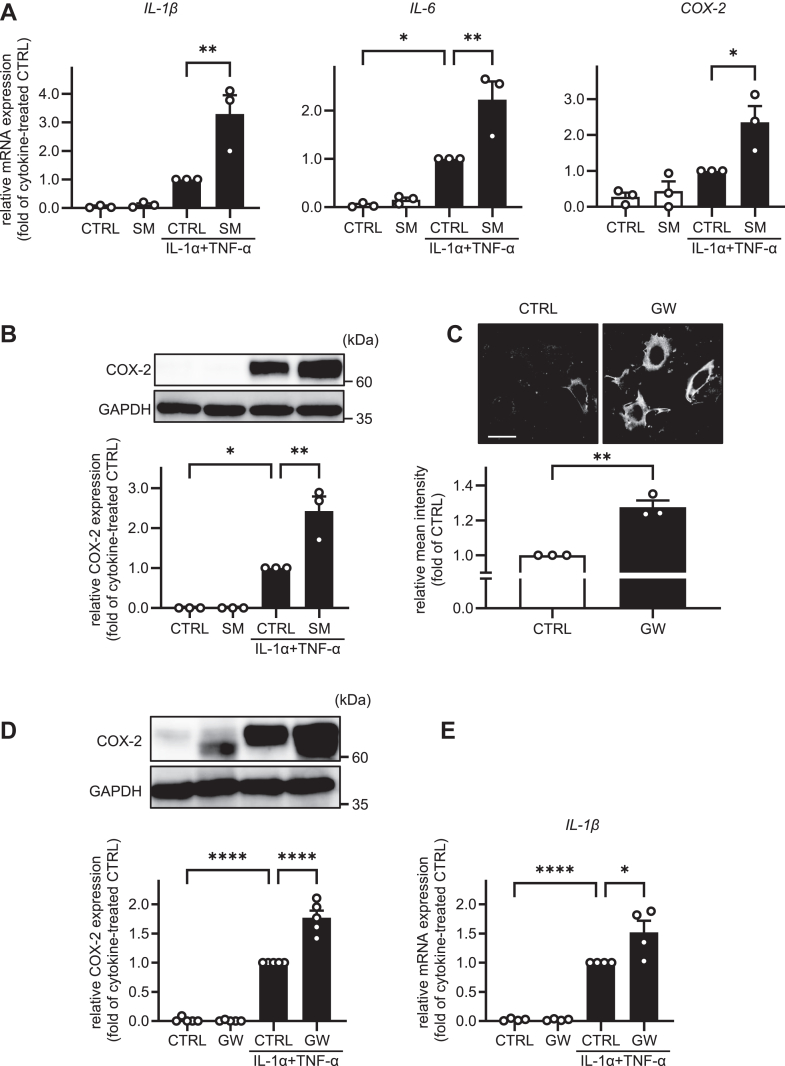
SM promotes astrocyte activation. A: HASTR/ci35 cells were treated with or without 1 μM SM for 48 h. The cells were treated with 3 ng/ml IL-1α, 50 ng/ml TNF-α, and SM for 24 h. The mRNA levels of *IL-1β*, *IL-6*, and *COX-2* were quantified by qPCR (n = 3). B: The protein levels of COX-2 were examined by western blotting (n = 3). C: HASTR/ci35 cells were treated with or without 10 μM GW4869 (GW) for 48 h. Plasma membrane SM levels were detected by lysenin staining (n = 3). D: HASTR/ci35 cells were treated with or without 10 μM GW4869 for 48 h. The cells were then incubated with 3 ng/ml IL-1α, 50 ng/ml TNF-α, and GW4869 for 24 h. The protein levels of COX-2 were examined by western blotting (n = 5). E: mRNA expression levels of *IL-1β* were analyzed by qPCR (n = 4). In (C), data are representative images of three independent experiments. All data are presented as mean ± SEM. A, B, D, and E, Tukey’s test. C, Student’s *t* test. ∗ *P* < 0.05 ∗∗*P <* 0.01 ∗∗∗∗ *P <* 0.0001. The scale bar shows 50 μm.

The effect of SM on astrocyte activation was assessed by examining the mRNA and protein expression levels of representative markers of astrocyte activation (*IL-1β*, *IL-6,* and *COX-2*). Treatment with IL-1α and TNF-α activates astrocytes (3, 4). In the present study, treatment of HASTR/ci35 cells with IL-1α/TNF-α for 24 h induced increases in the mRNA levels of *IL-1β*, *IL-6,* and *COX-2*, as well as the protein levels of COX-2. To assess the effects of SM on astrocyte activity, we used SM derived from bovine spinal cord. LC-MS/MS analysis showed that bovine spinal cord SM consisted primarily of d18:1/C24:1-SM (68.57%) and d18:1/C22:0-SM (22.40%), with minor components of d18:1/C20:0-SM (3.27%) and d18:1/C18:0-SM (5.76%) (Supplemental Fig. S1). Pretreatment of the cells with apparent 1 μM SM from bovine spinal cord for 48 h significantly enhanced the IL-1α/TNF-α induction of the markers of astrocyte activation (Fig. 1A, B). Next, we evaluated the effect of endogenous SM on astrocyte activation. It appeared from the TLC that GW4869, an inhibitor of neutral SMase, a plasma membrane-associated enzyme, significantly increased cellular SM levels in astrocytes treated for 48 h (Supplemental Fig. 2A). SM standards were applied to the TLC plate in increasing amounts to confirm the linearity of the signal. As shown in Supplemental Fig. 3A, B, the intensity of the SM spots increased in a dose-dependent manner and exhibited good linearity (*R*^2^ = 0.8579). To determine whether SM levels in the plasma membrane were altered by GW4869 treatment, cells were incubated with the SM-binding probe lysenin, followed by incubation with anti-lysenin antibody. The immunofluorescence images in Fig. 1C show that the treatment with GW4869 significantly increased SM intensity at the plasma membrane. Under these conditions, we determined whether increased endogenous SM levels affected the activation of astrocytes. As shown in Fig. 1D, the protein expression levels of COX-2 were significantly increased by GW4869 treatment in IL-1α/TNF-α-treated cells. Moreover, GW4869 treatment significantly increased *IL-1β* mRNA expression levels (Fig. 1E). These results suggest that SM, notably plasma membrane SM, promotes IL-1α/TNF-α-induced astrocyte activation.

### Knockdown of sphingomyelin synthase 1 (SMS1) and SMS2 suppresses astrocyte activation

**Fig. 2:**
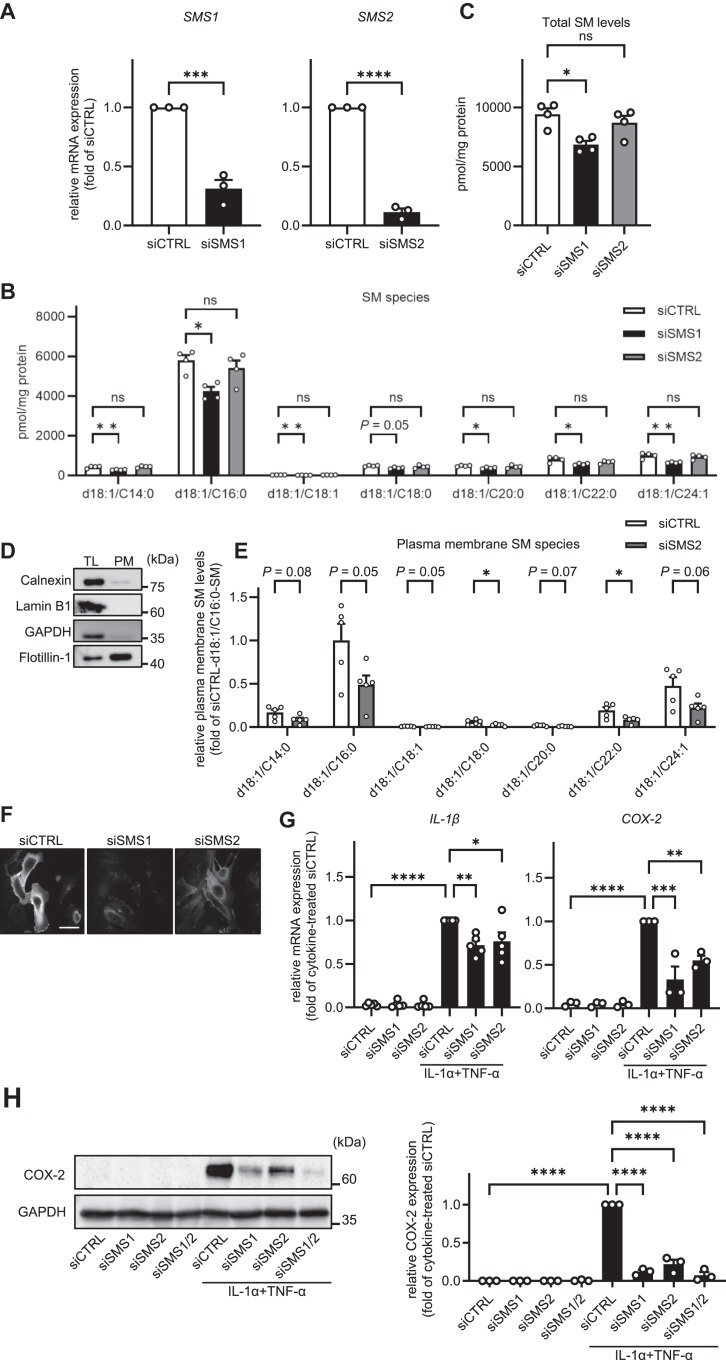
Knockdown of sphingomyelin synthase 1 (SMS1) and SMS2 suppresses astrocyte activation. A: HASTR/ci35 cells were transfected with control siRNA (siCTRL) or siRNA against SMS1 (siSMS1) or SMS2 (siSMS2) and incubated for 48 h. The knockdown efficiency of SMS1 or SMS2 was confirmed by qPCR (n = 3). B: Intracellular individual SM levels in SMS1- or SMS2- knockdown cells were quantified by LC-MS/MS analysis (n = 4). C: Total cellular SM levels were quantified by LC-MS/MS analysis (n = 4). D: The plasma membrane fraction was isolated using a plasma membrane extraction kit. Protein expression of calnexin (ER marker), lamin B1 (nuclear marker), GAPDH (cytosolic marker), and flotillin-1 (plasma membrane marker) was examined in total lysate (TL) and plasma membrane fraction (PM) by western blotting. E: SM levels in the plasma membrane extract were quantified by LC-MS/MS-based lipidomics. Individual SM levels were normalized to flotillin-1 intensity (Supplemental Fig. S5) (n = 5). F: HASTR/ci35 cells were transfected with control siCTRL, siSMS1, or siSMS2 and incubated for 48 h. Plasma membrane SM levels were analyzed by lysenin staining. G: The cells were further incubated with 3 ng/ml IL-1α and 50 ng/ml TNF-α. The mRNA levels of *IL-1β* and *COX-2* were quantified by qPCR (n = 3∼5). H: The protein levels of COX-2 were examined by western blotting (n = 3). All data are presented as mean ± SEM. In F, data are representative images of three independent experiments. A, E, Student’s *t* test. B, C, G, and H, Tukey’s test. ∗ *P* < 0.05 ∗∗*P* < 0.01 ∗∗∗*P* < 0.001 ∗∗∗∗ *P* < 0.0001. ns, not significant. The scale bar shows 50 μm.

SM is mainly synthesized in the trans-Golgi apparatus and plasma membrane by SMS1 and SMS2, respectively (9). To further examine the effect of SM on astrocyte activation, we analyzed the mRNA and protein expression levels of the markers of astrocyte activation in SMS1 and/or SMS2-knockdown cells. The knockdown efficiency of SMS1 and SMS2 was confirmed by measuring SMS1 and SMS2 mRNA expression levels. As shown in Figure 2A, *SMS1* and *SMS2* mRNA expression levels significantly decreased after siRNA treatment for 48 h. Next, we examined endogenous SM levels in SMS1 or SMS2 knockdown cells using the TLC assay. Interestingly, it appeared from the TLC that intracellular SM levels were significantly reduced by SMS1 knockdown, whereas SMS2 knockdown did not affect the intracellular SM content (Supplemental Fig. 2B). However, because interfering lipids such as lysophospholipids and glycerophospholipids can overlap with SM spots on TLC plates due to their polar headgroups, we next performed LC-MS/MS-based lipidomic analysis, which provides higher quantitative accuracy than TLC, to determine whether SMS1 or SMS2 knockdown alters the levels of individual SM species. As shown in Figure 2B, C, SMS1 knockdown reduced the levels of d18:0/C14:0-, d18:1/C16:0-, d18:1/C18:1-, d18:1/C20:0-, d18:1/C22:0-, d18:1/C24:1-SM, as well as total SM. In contrast, SMS2 knockdown had only a minor effect on individual SM species and total SM levels. Unexpectedly, SMS1 and SMS2 knockdown slightly affected cellular ceramide levels under these experimental conditions (Supplemental Fig. S4). Because several studies have reported that SMS2 locally generates SM, particularly at the plasma membrane (9, 10), we hypothesized that SMS2 knockdown would specifically reduce SM levels at the plasma membrane rather than throughout the whole cell. To test this, we isolated the plasma membrane fraction and conducted LC-MS/MS-based lipidomic analysis. As shown in Figure 2D, Western blotting confirmed the successful isolation of the plasma membrane, with flotillin-1 detected as a plasma membrane marker and no signals observed for other organelle markers. Subsequent LC-MS/MS analysis of the isolated fraction demonstrated that SMS2 knockdown markedly reduced individual SM species, indicating a specific decrease in SM levels at the plasma membrane (Fig. 2E). The signal intensity of flotillin-1 acquired by western blotting was used to normalize the levels of individual SM species (Supplemental Fig. S5). To further determine whether plasma membrane SM levels were altered in SMS1 or SMS2 knockdown cells, we assessed the plasma membrane SM content using lysenin staining. As shown in Figure 2F, knockdown of SMS1 attenuated the fluorescence intensity in whole cells, while the knockdown of SMS2 attenuated the intensity in the plasma membrane. These results indicate that SMS1 is responsible for most intracellular SM synthesis, whereas SMS2 locally synthesizes SM in the plasma membrane. Finally, we confirmed the effects of SMS1 and SMS2 knockdown on astrocyte activation. As shown in Figure 2G, H, SMS1 and/or SMS2 knockdown significantly reduced the IL-1α/TNF-α-induced expression of *IL-1β* and *COX-2* mRNA, as well as expression of COX-2 protein. These results suggest that the reduction of plasma membrane SM levels attenuates IL-1α/TNF-α-induced astrocyte activation.

### Knockdown of CERT suppresses astrocyte activation

**Fig. 3:**
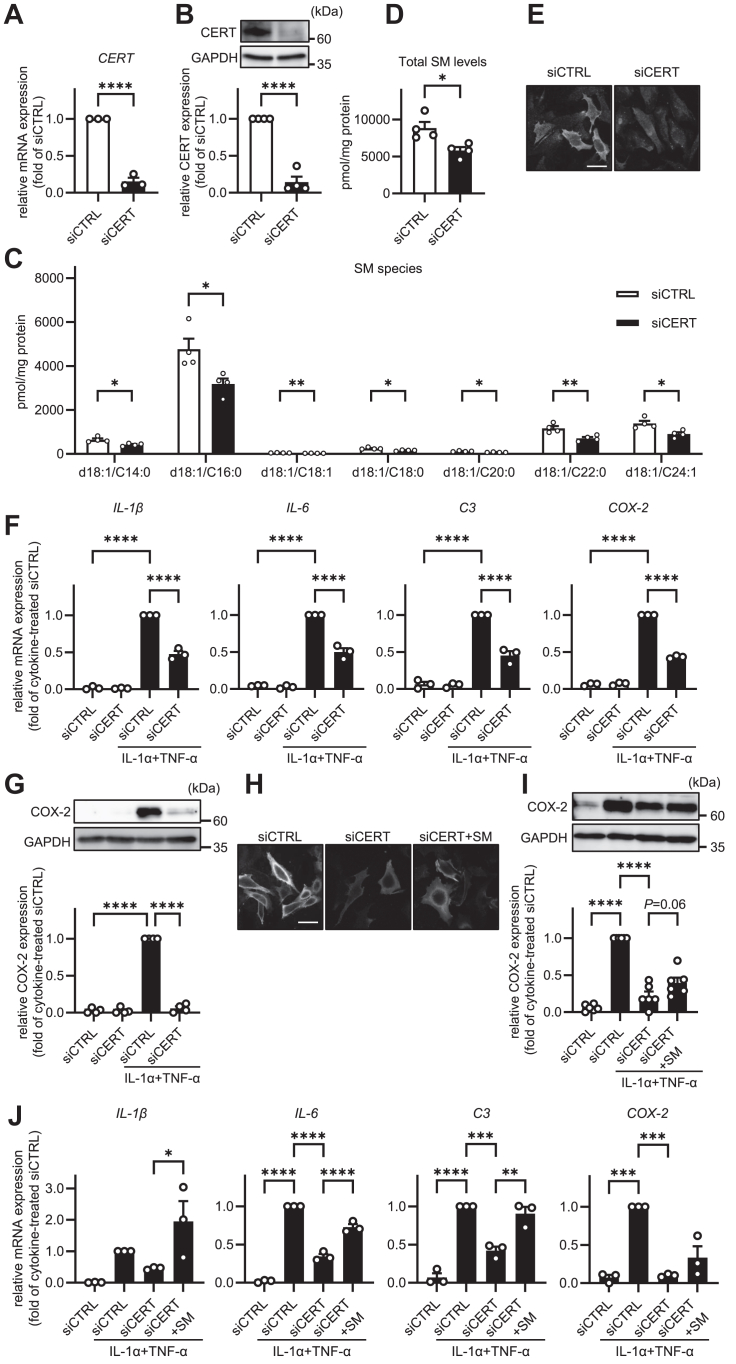
Knockdown of CERT suppresses astrocyte activation. A and B: HASTR/ci35 cells were transfected with control siRNA (siCTRL) or siRNA against CERT (siCERT) and incubated for 48 h. *CERT* mRNA expression and protein levels were quantified by qPCR and western blotting (n = 3∼4). C: Intracellular individual SM levels in CERT-knockdown cells were quantified by LC-MS/MS lipidomics (n = 4). D: Total cellular SM levels in CERT-knockdown cells were quantified by LC-MS/MS analysis (n = 4). E: Plasma membrane SM levels were analyzed by lysenin staining. F: The cells were further incubated with 3 ng/ml IL-1α and 50 ng/ml TNF-α for 24 h. The mRNA levels of *IL-1β*, *IL-6*, *C3*, and *COX-2* were quantified by qPCR (n = 3). G: The protein levels of COX-2 were examined by western blotting (n = 4). H: CERT knockdown cells were then treated with 1 μM SM for 1 h. Plasma membrane SM amounts were estimated by lysenin staining. I: SM-added cells were further incubated with 3 ng/ml IL-1α and 50 ng/ml TNF-α for 24 h. The protein levels of COX-2 were examined by western blotting (n = 6). J: The mRNA levels of *IL-1β*, *IL-6*, *C3*, and *COX-2* were quantified by qPCR (n = 3). In E and H, data are representative images of three independent experiments. All data are presented as mean ± SEM. A, B, C, and D, Student’s *t* test. F, G, I, and J, Tukey’s test. ∗ *P* < 0.05 ∗∗*P* < 0.01 ∗∗∗*P* < 0.001 ∗∗∗∗ *P* < 0.0001. The scale bar shows 50 μm.

To evaluate the effects of SM depletion on astrocyte activation, we next focused on the ceramide transport system. Ceramides synthesized in the ER are transported to the Golgi apparatus by CERT. Because SM synthesized by SMS1 depends on CERT-mediated ceramide transport, CERT inhibition leads to SM reduction without ceramide accumulation (21, 22). We examined the efficiency of CERT knockdown by measuring *CERT* mRNA and protein expression levels. These levels were reduced by treatment with CERT siRNA (Fig. 3A, B). To assess the effects of CERT knockdown on endogenous SM levels, we performed TLC analysis. As shown in Supplemental Fig. S2C, it appeared from the TLC that endogenous SM levels were significantly reduced in CERT knockdown cells. To reveal the effects of CERT knockdown on intracellular SM species levels, we performed LC-MS/MS analysis. As shown in Figure 3C, D, CERT knockdown significantly decreased the levels of several SM species (d18:0/C14:0, d18:1/C16:0, d18:1/C18:1, d18:1/C18:0, d18:1/C20:0, d18:1/C22:0, d18:1/C24:1), as well as total SM levels. In contrast, cellular ceramide levels were slightly affected by CERT knockdown (Supplemental Fig. S6). Furthermore, plasma membrane SM levels significantly decreased (Fig. 3E). Next, we examined the effects of CERT knockdown on the mRNA and protein expression of the markers of astrocyte activation. As shown in Figures 3F, G, CERT knockdown significantly reduced the IL-1α/TNF-α-induced mRNA levels of *IL-1β, IL-6, C3,* and *COX-2*, as well as the levels of COX-2 protein. These results suggest that SM reduction mediated by CERT knockdown suppresses astrocyte activation. Next, we evaluated the changes in astrocyte activation when the SM levels were rescued by the exogenous addition of SM after CERT knockdown. To analyze plasma membrane SM levels, we performed lysenin staining. As shown in Fig. 3H, the plasma membrane SM levels were remarkably decreased by CERT knockdown, while the addition of bovine spinal cord SM partly rescued SM levels. The extent of SM restoration is quantified in Supplemental Fig. S7. Under these conditions, we determined whether the exogenously rescued SM affected the activation of astrocytes. As shown in Figure 3I, J, astrocyte activation marker mRNA and COX-2 protein expression levels, which were decreased by the knockdown of CERT, were recovered by exogenously rescued bovine spinal SM. Since bovine SM contains various SM species, including d18:1/18:0, d18:1/C20:0, d18:1/C22:0, and d18:1/C24:1-SM (Supplemental Fig. S1), we next investigated which species play a pivotal role in astrocyte activity. Surprisingly, treatment with 1 μM individual SM species-d18:1/18:0, d18:1/C22:0, or d18:1/C24:1 did not recover astrocyte activity in CERT-knockdown cells (Supplemental Fig. S8A). Similarly, treatment with 1 or 3 μM d18:1/C16:0-SM, the most abundant SM species in HASTR/ci35 cells (Figs. 2B and 3C), did not recover its activity (Supplemental Fig. 8B). These findings supported the possibility that the presence of multiple SM species, rather than a single species, is critical for recovering astrocyte activity. These results suggest that SM, especially plasma membrane SM, promotes astrocyte activation.

### HASTR/ci35 cells are activated by IL-1α/TNF-α stimulation via the NF-κB pathway

**Fig. 4:**
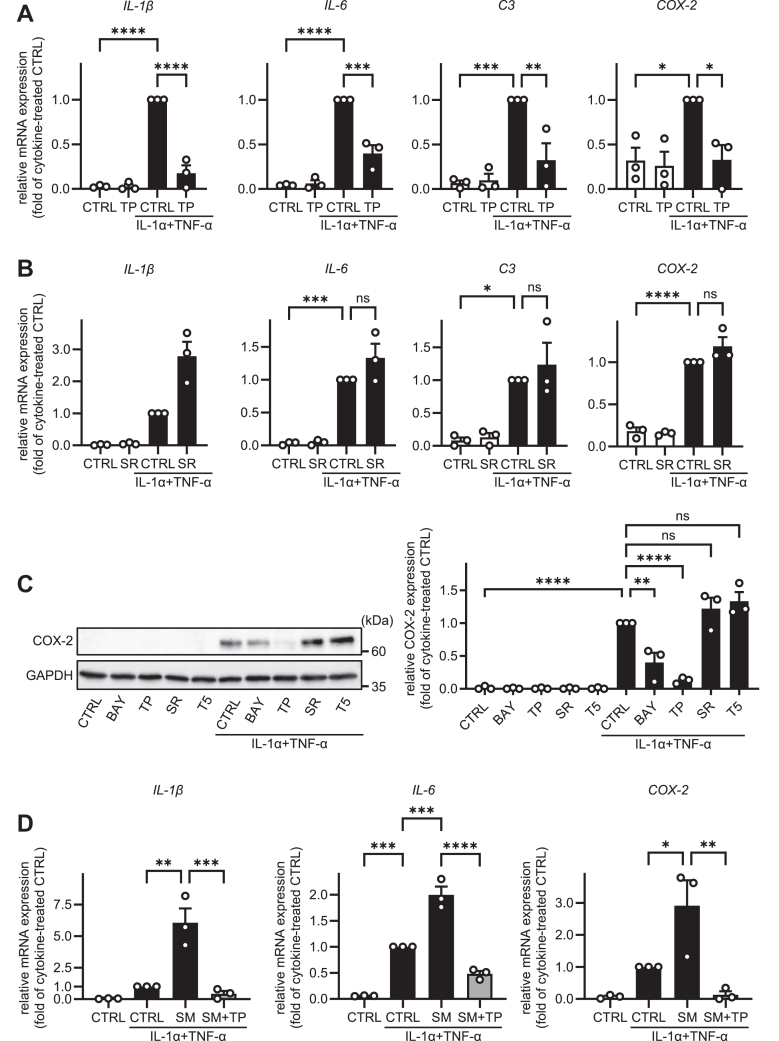
HASTR/ci35 cells are activated by IL-1α/TNF-α stimulation via the NF-κB pathway. A and B: HASTR/ci35 cells were pre-treated with 1 μM TPCA-1 (TP) or 1 μM SR11302 (SR) for 2 h in SFAM. The cells were then incubated with 3 ng/ml IL-1α and 50 ng/ml TNF-α for 24 h. The mRNA levels of *IL-1β*, *IL-6*, *C3* and *COX-2* were quantified by qPCR (n = 3). C: HASTR/ci35 cells were pre-treated with IKK inhibitors (1 μM BAY 11–7082 (BAY), 1 μM TP) or AP-1 inhibitors (1 μM SR, 10 μM T-5224 (T5)) for 2 h in SFAM. The cells were then incubated with 3 ng/ml IL-1α and 50 ng/ml TNF-α for 24 h. The protein levels of COX-2 were examined by western blotting (n = 3). D: HASTR/ci35 cells were treated with SM for 48 h, and then the cells were pre-incubated with 1 μM TPCA-1 for 2 h. The cells were further incubated with 3 ng/ml IL-1α, 50 ng/ml TNF-α, and 1 μM SM for 24 h. The mRNA levels of *IL-1β*, *IL-6*, and *COX-2* were analyzed by qPCR (n = 3). All data are presented as mean ± SEM. All data were analyzed using Tukey’s test. ∗ *P* < 0.05 ∗∗*P* < 0.01 ∗∗∗*P* < 0.001 ∗∗∗∗ *P* < 0.0001. ns, not significant.

Astrocytes are activated predominantly via the NF-κB and MAPK pathways (4, 5). To determine whether HASTR/ci35 human astrocytes cells are activated via these pathways, we measured *IL-1β*, *IL-6*, *C3*, and *COX-2* mRNA expression levels in cells treated with inhibitor of NF-κB or activator protein-1, AP-1. As shown in Figure 4A, B, the treatment with NF-κB inhibitor (TPCA-1; TP), but not AP-1 inhibitor (SR11302; SR), reduced the IL-1α/TNF-α-induced *IL-1β*, *IL-6*, *C3*, and *COX-2* mRNA expression levels. Concordantly, COX-2 protein expression levels were also decreased by treatment with NF-κB inhibitors (TP and BAY-7082; BAY), but not by treatment with AP-1 inhibitors (SR and T-5224; T5) (Fig. 4C). Consistent with these results, treatment with NF-κB inhibitors prevented SM-induced astrocyte activation (Fig. 4D). These results indicate that HASTR/ci35 is mainly activated via the NF-κB pathway, and SM-induced astrocyte activation is also mediated by this pathway.

### p65 translocation is not inhibited by CERT knockdown

**Fig. 5:**
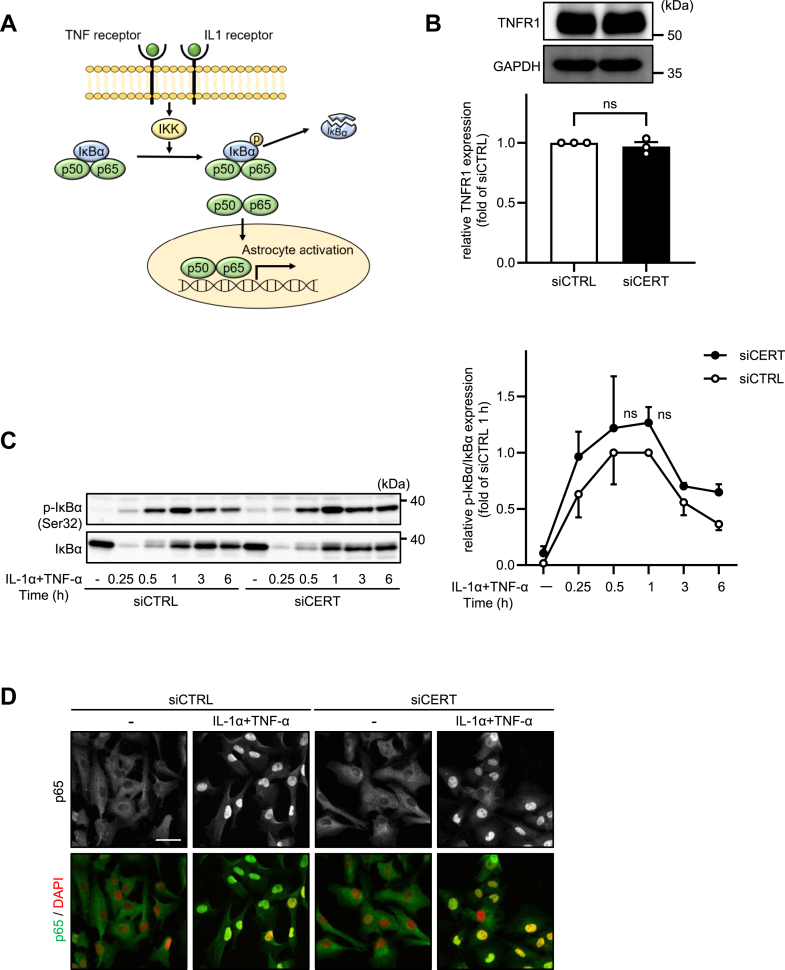
p65 translocation is not inhibited by CERT knockdown. A: Mechanism of NF-κB pathway activation. B: HASTR/ci35 cells were transfected with control siRNA (siCTRL) or siRNA against CERT (siCERT) and incubated for 48 h. TNF receptor 1 protein expression levels were analyzed by western blotting (n = 3). C: The cells were further incubated with 3 ng/ml IL-1α and 50 ng/ml TNF-α for 15 min to 6 h. The protein levels of IκBα and phospho-IκBα (p-IκBα) were examined by western blotting (n = 3). D: The cells were further incubated with 3 ng/ml IL-1α and 50 ng/ml TNF-α for 15 min. The cells were fixed and immunostained for p65. Typical images were shown (n = 3). B, Student’s *t* test. C, Tukey’s test. All data are presented as mean ± SEM. ns, not significant.

IL-1α/TNF-α-induced NF-κB signaling is initiated by specific phosphorylation of the IκB by the IκB kinase (IKK) complex. Subsequently, phosphorylated IκB proteins are rapidly ubiquitinated and degraded by proteasomes, which allows the NF-κB complex to translocate into the nucleus (Fig. 5A). SM is abundant in lipid rafts and provides a scaffold for several receptors, such as Toll-like receptors (23). Thus, we determined whether CERT knockdown can affect cytokine receptor protein expression by Western blot analysis. As shown in Figure 5B, TNFR1 protein expression levels did not change in CERT knockdown cells. To assess the effects of CERT knockdown on astrocyte activation, we investigated the changes in IκBα phosphorylation and degradation. These were not changed by CERT knockdown (Fig. 5C). Next, we examined the intracellular localization of p65 by immunostaining. As shown in Figure 5D, knockdown of CERT did not alter the IL-1α/TNF-α-induced nuclear translocation of p65 in cells. These results indicate that the knockdown of CERT did not alter the NF-κB signal transduction, at least until p65 nuclear translocation in cells.

### CERT knockdown reduces p65 acetylation

**Fig. 6:**
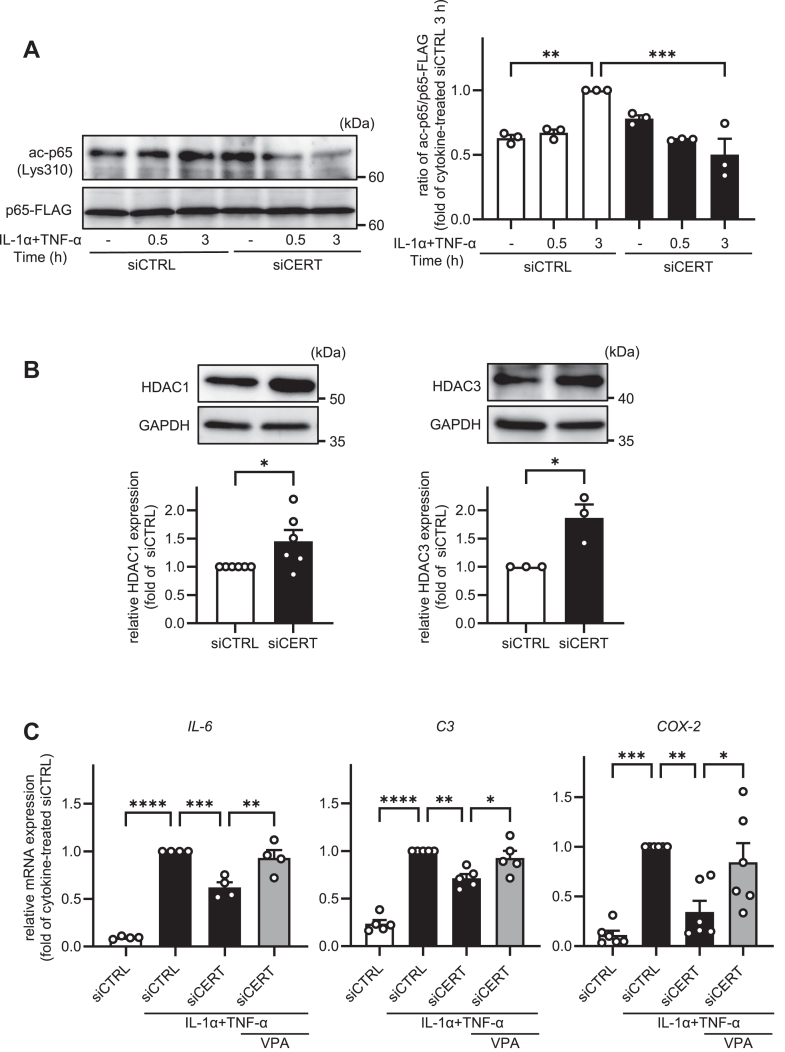
CERT knockdown reduces p65 acetylation. A: HASTR/ci35 cells were transfected with p65-FLAG plasmids. After 3 h, the cells were washed out and transfected with control siRNA (siCTRL) or siRNA against CERT (siCERT) and cultured for 48 h. The cells were further incubated with 3 ng/ml IL-1α and 50 ng/ml TNF-α for 30 min to 3 h. The protein levels of p65-FLAG and acetylated p65 were analyzed by western blotting (n = 3). B: HASTR/ci35 cells were transfected with control siRNA or siRNA against CERT and incubated for 48 h (n = 3). The protein levels of HDAC1 and HDAC3 were examined by western blotting (n = 3∼6). C: The cells were further pre-incubated with 3 mM valproic acid (VPA) for 30 min and then treated with 3 ng/ml IL-1α and 50 ng/ml TNF-α for 24 h. The mRNA levels of *IL-6*, *C3*, and *COX-2* were quantified by qPCR (n = 4∼6). All data are presented as mean ± SEM. A and C, Tukey’s test. B, Student’s *t* test. ∗ *P* < 0.05 ∗∗*P* < 0.01 ∗∗∗*P <* 0.001 ∗∗∗∗ *P* < 0.0001.

Various post-transcriptional modifications, such as phosphorylation, acetylation, and methylation, alter p65 transcription activity (24, 25). To determine whether CERT knockdown affects p65 post-transcriptional modifications, we first examined phosphorylated p65 protein expression in CERT knockdown cells. CERT knockdown did not change the IL-1α/TNF-α-induced phosphorylation at Ser536 of p65 (Supplemental Fig. S9). Interestingly, the knockdown of CERT significantly decreased the IL-1α/TNF-α-induced acetylation at Lys310 of p65 (Fig. 6A). Acetylation of p65 is regulated by histone acetyltransferases (HATs) and histone deacetylases (HDACs). The p300 HAT protein and the HDAC1 and HDAC3 HDACs are involved in regulating p65 acetylation (26, 27, 28). Thus, we examined changes in HDAC1 and HDAC3 protein expression levels. Notably, these levels were significantly increased following CERT knockdown (Fig. 6B), whereas p300 protein expression levels did not change (data not shown). Finally, to determine whether the suppression of astrocyte activation by CERT knockdown was mediated by HDAC1 and HDAC3, we examined the mRNA expression levels of astrocyte activation markers after treatment with the HDAC inhibitor valproic acid (VPA). As shown in Figure 6C, treatment with 3 mM VPA canceled the CERT knockdown-induced suppression of *IL-6*, *C3*, and *COX-2* mRNA levels. These results suggest that CERT knockdown-induced suppression of astrocyte activation is mediated by the induction of HDAC1 and HDAC3 protein expression, which results in the reduction of acetylated p65 (Lys310) protein expression and inhibition of the NF-κB pathway.

## Discussion

In the present study, we investigated the effects of altered cellular SM levels on astrocytic activity. Increasing cellular SM levels by exogenous addition of SM or inhibiting SM hydrolysis with GW4869 treatment enhanced the IL-1α/TNF-α-induced activation of astrocytes (Fig. 1). Conversely, decreasing cellular SM levels by knockdown of SMS1 and SMS2 attenuated the IL-1α/TNF-α-induced activation of astrocytes (Fig. 2). These results suggest that SM promotes the IL-1α/TNF-α-induced astrocyte activation. We next demonstrated that reducing cellular SM content without any increase in ceramide levels attenuated the IL-1α/TNF-α-induced activation of astrocytes (Fig. 3). Moreover, the decreased astrocytic activity after CERT knockdown was reversed by exogenously rescued SM. Thus, we conclude that SM plays an essential role in the regulation of astrocyte activity. However, in this study, SM levels were assessed in whole-cell lysates of GW4869-treated or SM-supplemented cells (Supplemental Figs. S2A and S7). Therefore, future studies are required to (i) identify specific subcellular sites (e.g., plasma membrane) where SM accumulation occurs using more quantitative approaches such as LC-MS/MS, and (ii) determine whether other sphingolipid metabolites are also altered.

SM is mainly synthesized in the trans-Golgi apparatus and plasma membrane by SMS1 and SMS2, respectively. SMS1 is involved in SM biosynthesis, while SMS2 is involved in remodeling of plasma membrane structure (29). Consistent with these observations, in the present study SMS1 knockdown reduced intracellular SM levels by approximately 50%, whereas SMS2 knockdown slightly changed intracellular SM levels (Supplemental Fig. 2B, C). However, the knockdown of SMS1 and SMS2 suppressed astrocyte activation. Considering that SMS2 is predominantly localized in the plasma membrane, these results suggest that SM in the plasma membrane plays a pivotal role in astrocyte activation. Indeed, SM levels in the plasma membrane were reduced following SMS2 knockdown (Fig. 2E, F).

SM interacts with lipids, such as cholesterol and phospholipids, to form characteristic lipid domains called lipid rafts (30). Lipid rafts are required for TNFR1 clustering and NF-κB signal transition, while their disruption leads to receptor destabilization (31, 32). Reduction of plasma membrane SM leads to TNFR1 internalization and attenuated TNF-α-induced IκBα degradation and p65 translocation into the nuclei of SMS2-deficient mouse primary macrophages (33). However, we observed that CERT knockdown did not affect TNFR1 total protein levels, IκBα degradation, and p65 nuclear translocation (Fig. 5), indicating that the SM levels in the plasma membrane do not affect the IL-1α/TNF-α-induced receptor signaling in our experimental conditions.

NF-κB signaling regulates the activity of astrocytes (4, 5). In the present study, SM-induced astrocyte activation was attenuated by treatment with an inhibitor of NF-κB (Fig. 4D). p65 acetylation is a reversible reaction regulated by HAT proteins (e.g., p300/CRB) and HDAC proteins (e.g., HDAC1/3 and NAD-dependent protein deacetylase sirtuin-1). Seven residues have been identified as acetylation sites in p65; all are lysine residues (Lys122, Lys123, Lys218, Lys221, Lys310, Lys314, and Lys315) (25). Specific acetylation of p65 lysine residues, particularly Lys310, is crucial for its transcriptional activity. Of note, the transcriptional activity of p65 is significantly reduced in the p65K310R mutant, which does not undergo acetylation (34, 35). In this study, the knockdown of CERT significantly decreased the IL-1α/TNF-α-induced acetylation at Lys310 of p65 via induction of HDAC1/3 (Fig. 6A, B). Bromodomains of Brd4, a coactivator of p65, reportedly directly bind to the acetylated Lys310 of p65 and promote NF-κB transcriptional activity in HEK293T and THP-1 cells (36). Moreover, treatment with (+)-JQ1, a BET bromodomain inhibitor that inhibits Brd4 activity, attenuates NF-κB transcriptional activity without inhibiting IκBα degradation and p65 nuclear translocation in INS 832/13 cells (37). Consistent with these reports, the present findings indicate that SM appears to regulate the transcriptional activity of p65 rather than its nuclear translocation.

We demonstrated that the knockdown of CERT induced HDAC1/3 protein expression, resulting in the maintenance of lower levels of acetylated-p65 (Lys310) and subsequent suppression of the NF-κB pathway in IL-1α/TNF-α-treated astrocytes (Fig. 6A, B). The underlying mechanisms by which CERT knockdown increases the protein expression of HDAC1/3 remain unclear. According to previous reports, the transforming growth factor-beta (TGF-β)/Smad signaling pathway promotes HDAC1/3 protein expression in HK-2 human renal cells (38). It has also been reported that TNF-α-induced NF-κB signaling inhibits the TGF-β/Smad signaling pathway (39). Thus, inhibiting NF-κB signaling by CERT knockdown may activate the TGF-β/Smad signaling pathway, resulting in the up-regulation of HDAC1/3. In addition to the transcriptional regulation of HDAC1/3, the knockdown of CERT may also regulate its degradation. Both HDAC1 and HDAC3 are degraded by the ubiquitin-proteasome system, which is mediated by specific ubiquitination caused by the mouse double minute 2 homolog and Siah E3 ubiquitin protein ligase 2, respectively (40, 41). The nuclear receptor co-repressor N-CoR prevents HDAC3 from proteasomal degradation by directly binding to its C-terminal domain, making it more stable (42). Thus, CERT knockdown may modulate those HDAC1/3 degradation mechanisms. However, the exact mechanism by which SM regulates HDAC1/3 expression requires further investigation.

In addition to astrocytes, microglia play a pivotal role in CNS inflammation (43, 44). To determine whether the SM-NF-κB signaling axis is specific to astrocytes or more broadly applicable, we evaluated the effects of SM addition or reduction in HMC3 cells, a human microglia-like cell line (detailed culture conditions are described in Supplementary method). As shown in Supplemental Fig. S10A, the addition of 1 μM SM increased the expression levels of *IL-1β* mRNA induced by IL-1α/TNF-α, similar to the effect observed in astrocytes. In contrast, as shown in Supplemental Fig. S10B, C, CERT knockdown decreased the expression levels of *IL-1β*, *TNF-α*, and *CCL2* mRNAs mediated by IL-1α/TNF-α stimulation. These results indicate that the SM-IL-1α/TNF-α-induced NF-κB signaling axis is not limited to astrocytes but also functions in microglia.

Since SM is the most abundant sphingolipid in mammalian cells, its reduction can influence other cellular sphingolipid metabolites, such as glycosphingolipids and ceramide. To determine whether sphingolipid metabolites affect astrocyte activity, we assessed the effects of inhibitors of sphingolipid metabolite enzymes on astrocyte activity (Supplemental Fig. 11A). As shown in Supplemental Fig. 11B, treatment with Eliglustat (Eli), an inhibitor of ceramide glucosyltransferase, did not alter the expression levels of *IL-1β* and *IL-6* mRNA. Similarly, COX-2 protein levels remained unchanged in eliglustat-treated cells (Supplemental Fig. 11C). Likewise, treatment with myriocin (Myr) or Fumonisin B1 (FB1), an inhibitor of serine palmitoyl-CoA transferase (SPT) and ceramide synthase (CERS), respectively, did not affect the mRNA expression levels of *C3*, *IL-1β*, and *IL-6* (Supplemental Fig. 11D). Furthermore, as mentioned above, CERT or SMS knockdown slightly altered endogenous ceramide levels (Supplemental Figs. S4 and S6). Taken together, these results suggest that SM reduction-induced alterations of other sphingolipids might slightly affect astrocyte activity.

Based on their gene expression profiles, astrocytes are divided into neurotoxic (commonly known as A1 astrocytes) and neuroprotective (A2 astrocytes) phenotypes. Although the NF-κB signaling pathway converts astrocytes to an A1 phenotype, recent findings indicate that it also converts astrocytes to an A2 phenotype (45). Interestingly, CERT knockdown suppressed A1 astrocyte gene expression (Fig. 3F), but did not alter A2 the mRNA expression levels (*SphK1 and PTX3*) (Supplemental Fig. 12A). These results suggest that SM reduction mediated by CERT inhibition might selectively inhibit differentiation to the A1 phenotype, but not the A2 phenotype, induced by IL-1α/TNF-α treatment in HASTR/ci35 cells. Moreover, treatment of HASTR/ci35 cells with NF-κB inhibitor (TPCA-1) or AP-1 inhibitor (SR11302) did not affect IL-1α/TNF-α-induced A2 astrocyte marker mRNA expression levels (Supplemental Fig. 12B, C). These results indicate that the phenotypic changes to A2 astrocytes mediated by IL-1α/TNF-α treatment are regulated by another signaling pathway(s). Further studies are needed to reveal how IL-1α/TNF-α stimulation regulates A2 astrocyte marker transcription.

Abnormal astrocyte activation has been observed in several neurodegenerative diseases, such as Alzheimer’s disease, multiple sclerosis, Parkinson’s disease, and Huntington’s disease (3, 46). Recent studies reported that the SM levels were significantly elevated in U251 astroglioma cells treated with Aβ1-42 oligomers, and that the treatment of Aβ1-42 oligomers can induce astrocyte activation (47, 48). Moreover, lipidomic analysis revealed that the levels of some SM species were increased in postmortem brains of patients with Alzheimer’s disease (49, 50). These reports imply that the SM species are involved in the pathology of Alzheimer’s disease via astrocyte activation.

In summary, sphingomyelin regulates IL-1α/TNF-α-induced astrocyte activation via the NF-κB pathway through the modulation of acetylated p65 levels. Our findings will be helpful in understanding the relationship between astrocyte activation and sphingolipid.

## Data availability

All data within the article are available upon request.

## Supplemental data

This article contains supplemental data.

## Conflict of interest

The authors declare no conflict of interest with the contents of this article.
