# Supplementary material for: Sphingomyelin regulates astrocyte activity by regulating NF-κB signaling via HDAC1/3 expression
Source: J Lipid Res. 2025 Nov 4;66(12):100933. doi: 10.1016/j.jlr.2025.100933 (PMC12721041; doi:10.1016/j.jlr.2025.100933)
Supplement: Supplementary Table S1 [file mmc1.docx]

**Supplementary Table 1**. The sequences of siRNAs used in this study.

| siRNA | Sequences (5’→3’) |
| --- | --- |
| siCTRL | anti-sense: UUGAUGUGUUUAGUCGCUAdT^†^dT  sense: UAG(M)^‡^CG(M)ACUAAACACAUCAAdTdT |
| siSMS1 | anti-sense: UAAGAUCGAGGUACAAUUCdTdT  sense: G(M)AAUUG(M)UACCUCG(M)AUCUUAdTdT |
| siSMS2 | anti-sense: UUUCAUUGGCCAUUGAAUGdTdT  sense: CAUUCAAUG(M)G(M)CCAAUG(M)AAAdTdT |
| siCERT | anti-sense: AAAUUUAGGAUACUCUCGCdTdT  sense: G(M)CG(M)AG(M)AG(M)UAUCCUAAAUUUdTdT |

†dT; deoxythymine. ‡G(M); 2'-oxymethylated guanosine.
