# Supplementary material for: Sphingomyelin regulates astrocyte activity by regulating NF-κB signaling via HDAC1/3 expression
Source: J Lipid Res. 2025 Nov 4;66(12):100933. doi: 10.1016/j.jlr.2025.100933 (PMC12721041; doi:10.1016/j.jlr.2025.100933)
Supplement: Supplementary Table S2 [file mmc2.docx]

**Supplementary Table 2.** Primer sequences used in this study.

| Primer name | Sequences (5’→3’) |
| --- | --- |
| *C3* | forward: AAAAGGGGCGCAACAAGTTC  reverse: GATGCCTTCCGGGTTCTCAA |
| *COX-2* | forward: CTTCACGCATCAGTTTTTCAAG  reverse: TCACCGTAAATATGATTTAAGTC  CAC |
| *GAPDH* | forward: GCACCGTCAAGGCTGAGAAC  reverse: TGGTGAAGACGCCAGTGGA |
| *IL-6* | forward: AGACAGCCACTCACCTCTTCAG  reverse: TTCTGCCAGTGCCTCTTTGCTG |
| *IL-1β* | forward: CCACAGACCTTCCAGGAGAATG  reverse: GTGCAGTTCAGTGATCGTACAGG |
| *SphK1* | forward: GCAGAAGTTGGCATGGTAGC  reverse: ACACACCTTTCCCATCCTTG |
| *PTX3* | forward: GTGGGTGGAGAGGAGAACAA  reverse: TTCCTCCCTCAGGAACAATG |
| *SMS1* | forward: CAACATTGGCGTAGACAT  reverse: TAGGAGGTACTCGTTCGTG |
| *SMS2* | forward: AGAAGTGACGAGGCGAAT  reverse: GATACAAGTCAATAGTGGGACG |
| *CERT* | forward: TGTGGATCATGACAGTGCTC  reverse: ATTTCCTGGTTTCCCTCTGG |
| *TNF-α* | forward: TCCTTCAGACACCCTCAACC  reverse: AGGCCCCAGTTTGAATTCTT |
| *CCL2* | forward: CAGCCAGATGCAATCAATGCC  reverse: TGGAATCCTGAACCCACTTCT |
