# Supplementary material for: Sphingomyelin regulates astrocyte activity by regulating NF-κB signaling via HDAC1/3 expression
Source: J Lipid Res. 2025 Nov 4;66(12):100933. doi: 10.1016/j.jlr.2025.100933 (PMC12721041; doi:10.1016/j.jlr.2025.100933)
Supplement: Supplementary Table S3 [file mmc3.docx]

**Supplementary Table 3.** The setting parameters of SCIEX QTRAP^®^4500 mass spectrometers for MRM detection of ceramide species.

| Ceramide species | Q1 m/z | Q3 m/z | DP (V) | CE (V) | CXP (V) |
| --- | --- | --- | --- | --- | --- |
| d18:1/C6:0-Cer | 398.2 | 380.3 | 56 | 13 | 14 |
| d18:1/C14:0-Cer | 510.6 | 264.4 | 55 | 35 | 15 |
| d18:1/C16:0-Cer | 538.7 | 264.4 | 55 | 37.5 | 15 |
| d18:1/C17:0-Cer | 552.7 | 264.4 | 55 | 37.5 | 15 |
| d18:1/C18:1-Cer | 564.7 | 264.4 | 55 | 37.5 | 15 |
| d18:1/C18:0-Cer | 566.7 | 264.4 | 55 | 37.5 | 15 |
| d18:1/C20:0-Cer | 594.7 | 264.4 | 55 | 37.5 | 15 |
| d18:1/C22:0-Cer | 622.8 | 264.4 | 55 | 37.5 | 15 |
| d18:1/C24:1-Cer | 648.9 | 264.4 | 55 | 42.5 | 15 |
