# Supplementary material for: Sphingomyelin regulates astrocyte activity by regulating NF-κB signaling via HDAC1/3 expression
Source: J Lipid Res. 2025 Nov 4;66(12):100933. doi: 10.1016/j.jlr.2025.100933 (PMC12721041; doi:10.1016/j.jlr.2025.100933)
Supplement: Supplementary Table S4 [file mmc4.docx]

| SM species | Q1 m/z | Q3 m/z | DP (V) | CE (V) | CXP (V) |
| --- | --- | --- | --- | --- | --- |
| d18:1/C12:0-SM | 647.7 | 184 | 81 | 45 | 16 |
| d18:1/C14:0-SM | 675.4 | 184 | 81 | 45 | 16 |
| d18:1/C16:0-SM | 703.8 | 184 | 81 | 45 | 16 |
| d18:1/C17:0-SM | 717.3 | 184 | 81 | 48.5 | 16 |
| d18:1/C18:1-SM | 729.7 | 184 | 81 | 48.5 | 16 |
| d18:1/C18:0-SM | 731.7 | 184 | 81 | 48.5 | 16 |
| d18:1/C20:0-SM | 759.6 | 184 | 81 | 53.5 | 16 |
| d18:1/C22:0-SM | 787.5 | 184 | 81 | 56 | 16 |
| d18:1/C24:1-SM | 813.8 | 184 | 81 | 56 | 16 |

**Supplementary Table 4.** The setting parameters of SCIEX QTRAP^®^4500 mass spectrometers for MRM detection of SM species.
