# Supplementary material for: Sphingomyelin regulates astrocyte activity by regulating NF-κB signaling via HDAC1/3 expression
Source: J Lipid Res. 2025 Nov 4;66(12):100933. doi: 10.1016/j.jlr.2025.100933 (PMC12721041; doi:10.1016/j.jlr.2025.100933)
Supplement: Supplementary Data 1 [file mmc5.pdf]

Supplementary information:

Sphingomyelin regulates astrocyte activity by regulating NF-κB signaling via HDAC1/3 expression

Ryo Kadowaki[1], Hana Hirose[1], Gai Takimoto[1], Takafumi Kohama[1], and Hiroyuki Nakamura[1, *]

[1] Laboratory of Chemical Pharmacology, Graduate School of Pharmaceutical Sciences, Chiba University, 1-8-1, Inohana, Chuo-ku, Chiba 260-8675, Japan.

Supplementary Figure 1

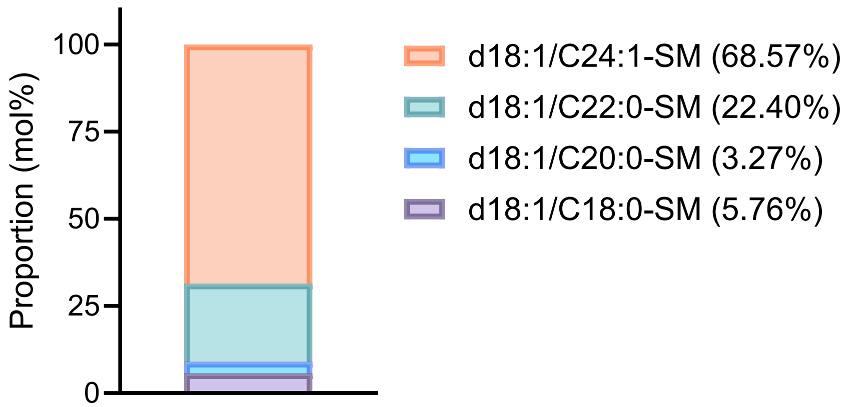

Supplementary Figure 1. A proportion of SM species in the bovine spinal cord SM

A composition of SM species in the bovine spinal cord-derived SM was analyzed by LC-MS/MS.

Supplementary Figure 2

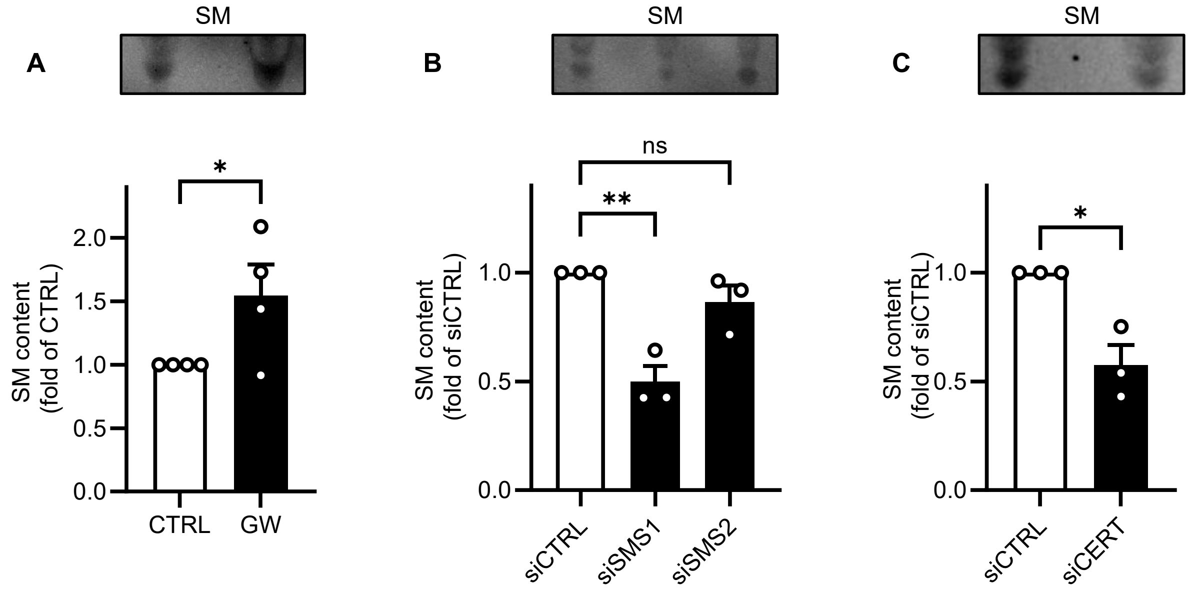

Supplementary Figure 2. Effects of GW4869 treatment or knockdown of SMS1, SMS2, or CERT on SM levels in HASTR/ci35 cells

(A) HASTR/ci35 cells were treated with or without 10 μM GW4869 (GW) for 48 h. Endogenous SM content was quantified by TLC assay (n=4). **(B)** HASTR/ci35 cells were transfected with control siRNA (siCTRL) or siRNA targeting SMS1 (siSMS1) or SMS2 (siSMS2) and incubated for 48 h. The amounts of SM were quantified by TLC assay (n=3). **(C)** HASTR/ci35 cells were transfected with siCTRL or siRNA targeting CERT (siCERT) and incubated for 48 h. The amounts of SM were quantified by TLC assay (n=3). All data are presented as mean ± SEM. A and C, Student's t-test. B, Tukey's test. * $P < 0.05$ ** $P < 0.01$. ns, not significant.

Supplementary Figure 3

A

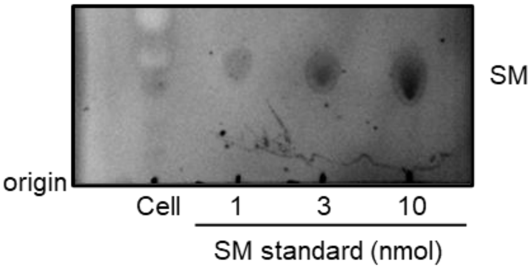

B

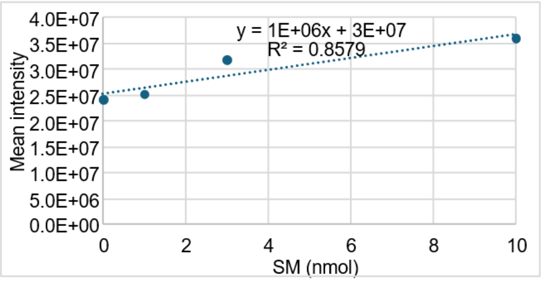

Supplementary Figure 3. Confirmation of SM signal at TLC

(A) Cultured HASTR/ci35 cells were washed twice with PBS and then incubated in SFAM for 48 h. Total lipids were extracted by the Bligh and Dyer method, as described in the *Materials and Methods* section. The extracted lipids were separated by TLC. SM standards were applied in a dose-dependent manner. **(B)** R^2 value is shown.

Supplementary Figure 4

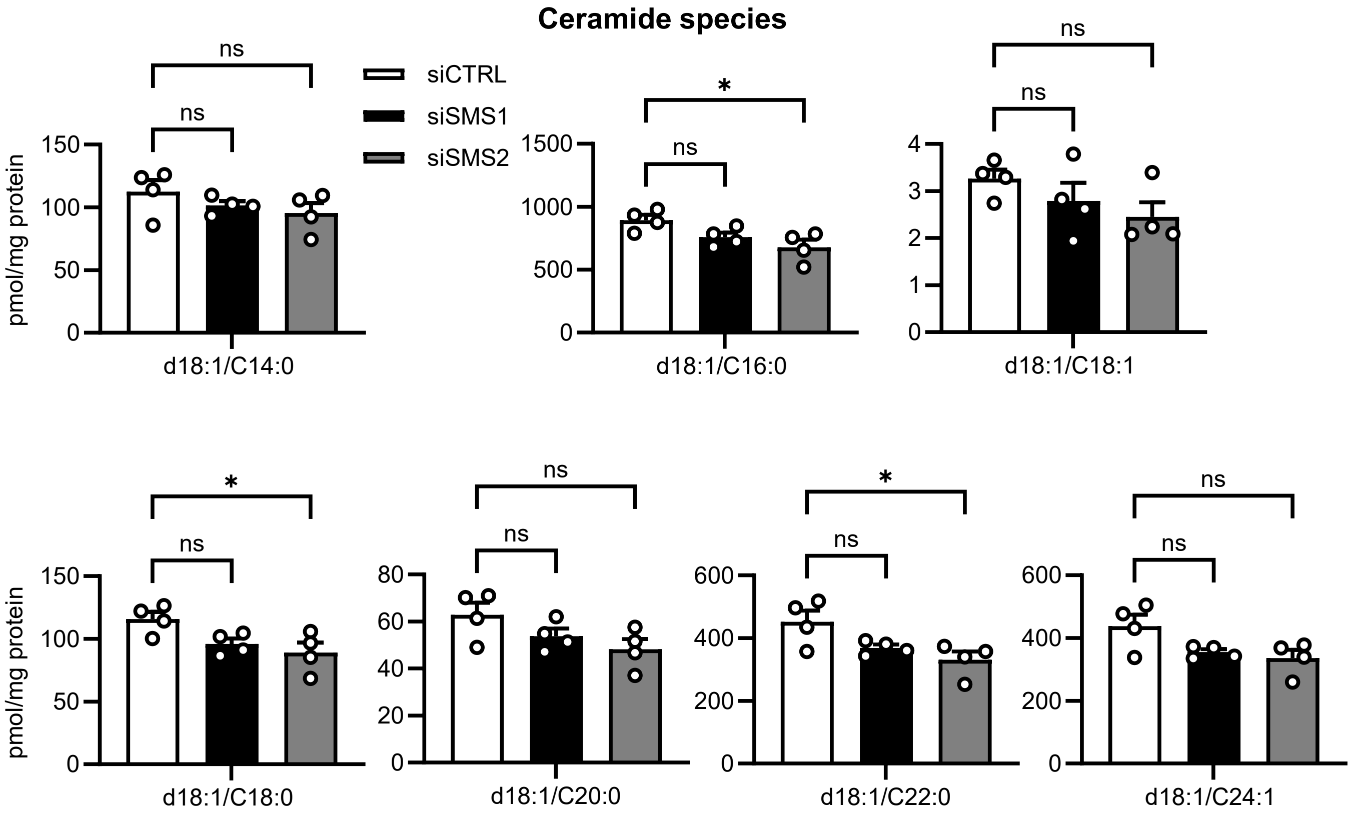

Supplementary Figure 4. The levels of SM and ceramide species in SMS-knockdown HASTR/ci35 cells

Cellular ceramide levels in SMS1 or SMS2-knockdown HASTR/ci35 cells were analyzed by LC-MS/MS-based lipidomics (n=4). All data are presented as mean ± SEM. All data were analyzed by Tukey's test. * *P* < 0.05. ns, not significant.

Supplementary Figure 5

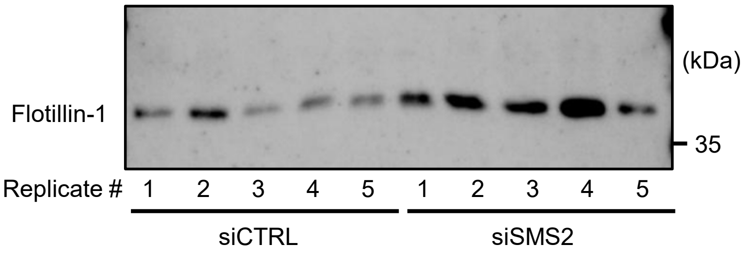

Supplementary Figure 5. Expression of flotillin-1 in the plasma membrane fraction of SMS2 knockdown cells

The plasma membrane fraction was isolated from cells treated with control siRNA (siCTRL) or SMS2 siRNA (siSMS2). Flotillin-1 expression was quantified by western blotting (n=5). The signal intensity of flotillin-1 was used to normalize the LC-MS/MS data shown in **Figure 2E**.

Supplementary Figure 6

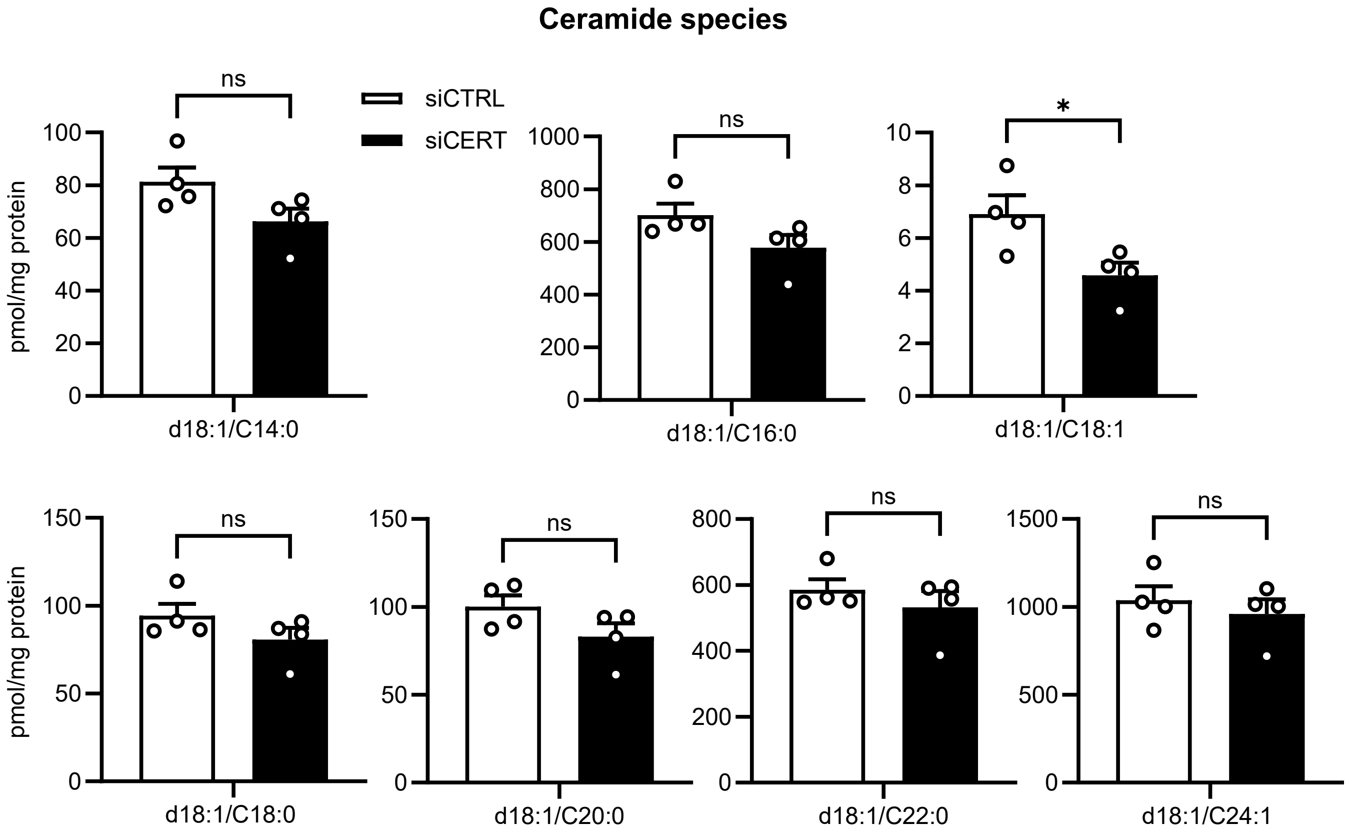

Supplementary Figure 6. The levels of SM and ceramide species in CERT-knockdown HASTR/ci35 cells

Cellular ceramide levels in CERT-knockdown HASTR/ci35 cells were analyzed by LC-MS/MS-based lipidomics (n=4). All data are presented as mean ± SEM. All data were analyzed by Student's t-test. * $P < 0.05$. ns, not significant.

Supplementary Figure 7

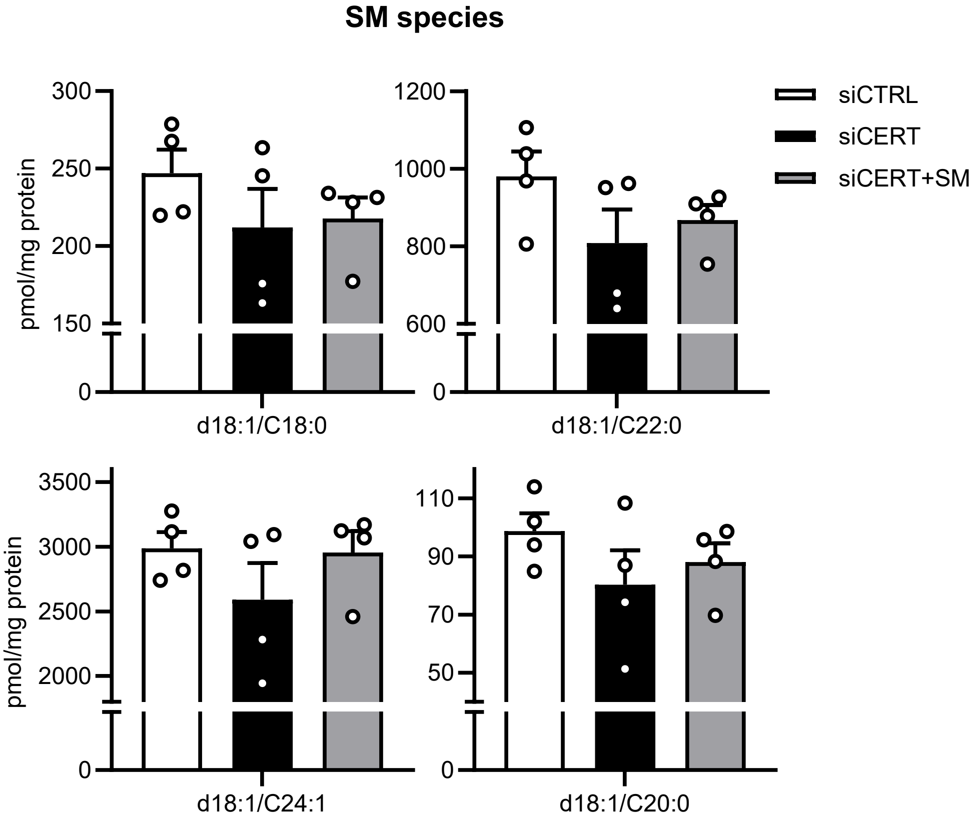

Supplementary Figure 7. SM levels in CERT knockdown cells supplemented with exogenous SM

Cells were transfected with control siRNA (siCTRL) or CERT siRNA (siCERT) for 48 h. CERT knockdown cells were then treated with 1 µM bovine spinal cord-derived SM for 1 h. The levels of d18:1/C18:0-, C20:0-, C22:0-, and C24:1-SM species were quantified by LC-MS/MS (n=4).

Supplementary Figure 8

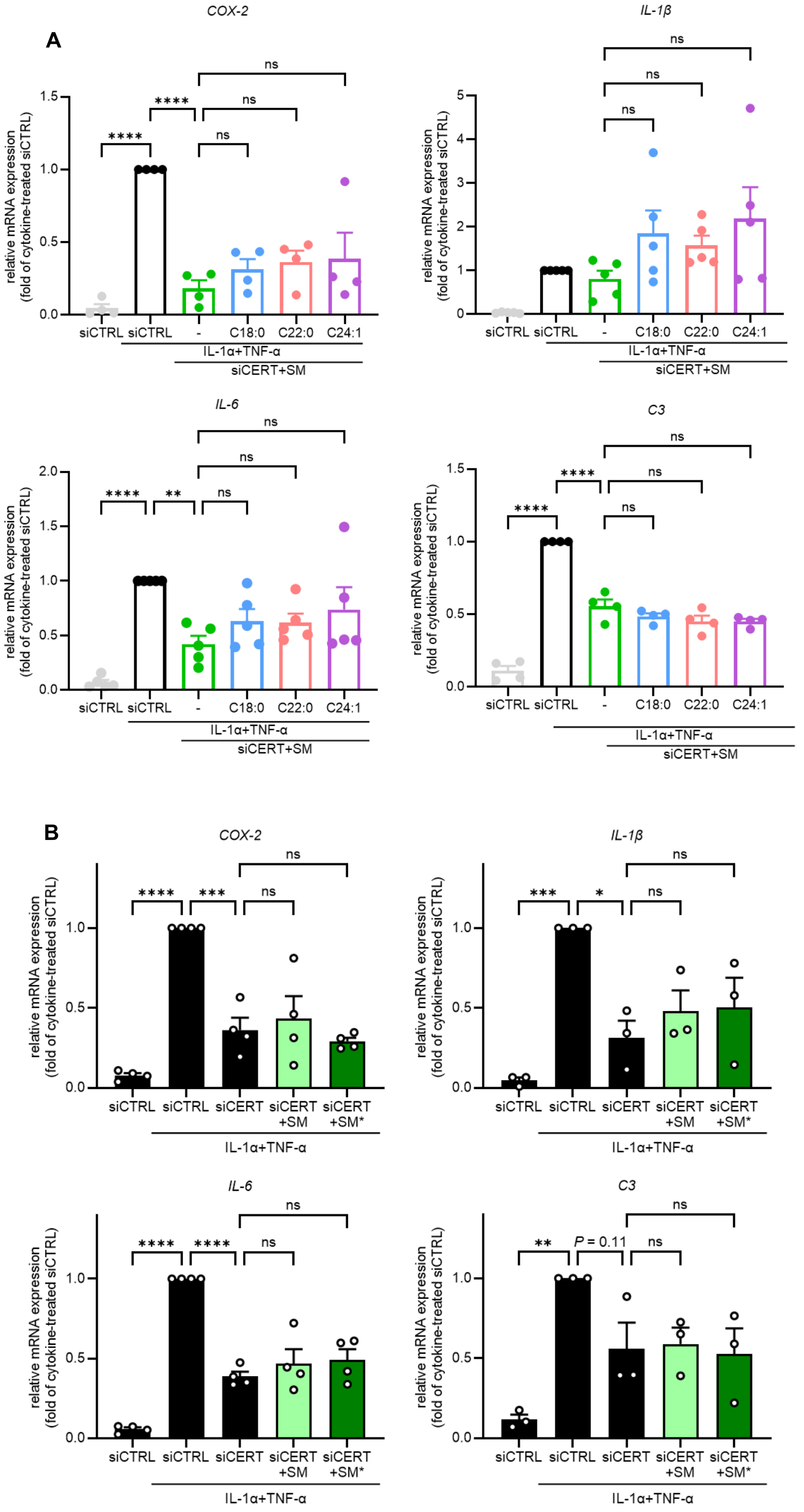

Supplementary Figure 8. A sole rescue of SM species did not recover IL-1α/TNF-α-induced HASTR/ci35 activation

HASTR/ci35 cells were treated with control siRNA (siCTRL) or siRNA against CERT (siCERT) for 48 h in SFAM. **(A)** The cells were further treated with 1 μM d18:1/C18:0-, d18:1/C22:0-, or d18:1/C24:1-SM for 1 h. Then, the cells were incubated with 3 ng/ml IL-1α and 50 ng/ml TNF-α for 24 h. The mRNA expression levels of representative astrocyte activation markers were quantified by qPCR (n=4~5). **(B)** The cells were further treated with 1 μM or 3 μM d18:1/C16:0-SM for 1 h. Then, the cells were incubated with 3 ng/ml IL-1α and 50 ng/ml TNF-α for 24 h. An asterisk shows 3 μM d18:1/C16:0-SM. The mRNA expression levels of representative astrocyte activation markers were quantified by qPCR (n=3~4). All data are presented as mean ± SEM. All data were analyzed by Tukey's test. * $P < 0.05$ ** $P < 0.01$ *** $P < 0.001$ **** $P < 0.0001$. ns, not significant.

Supplementary Figure 9

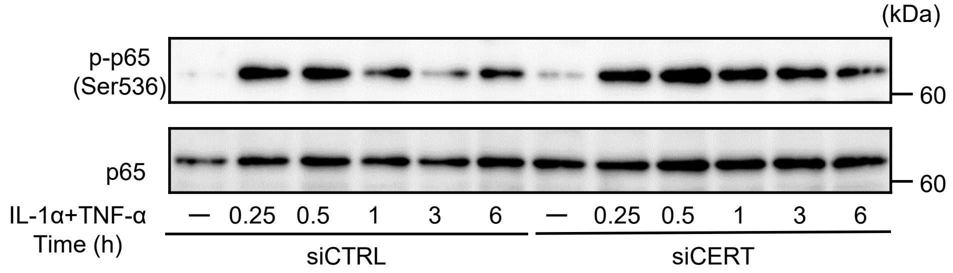

Supplementary Figure 9. p65 phosphorylation does not change by CERT knockdown

HASTR/ci35 cells were transfected with control siRNA (siCTRL) or siRNA against CERT (siCERT) and incubated for 48 h. The cells were further incubated with 3 ng/ml IL-1α and 50 ng/ml TNF-α for 15 min to 6 h. The protein levels of p65 and phospho-p65 (p-p65^{ser536}) were examined by western blotting.

Supplementary Figure 10

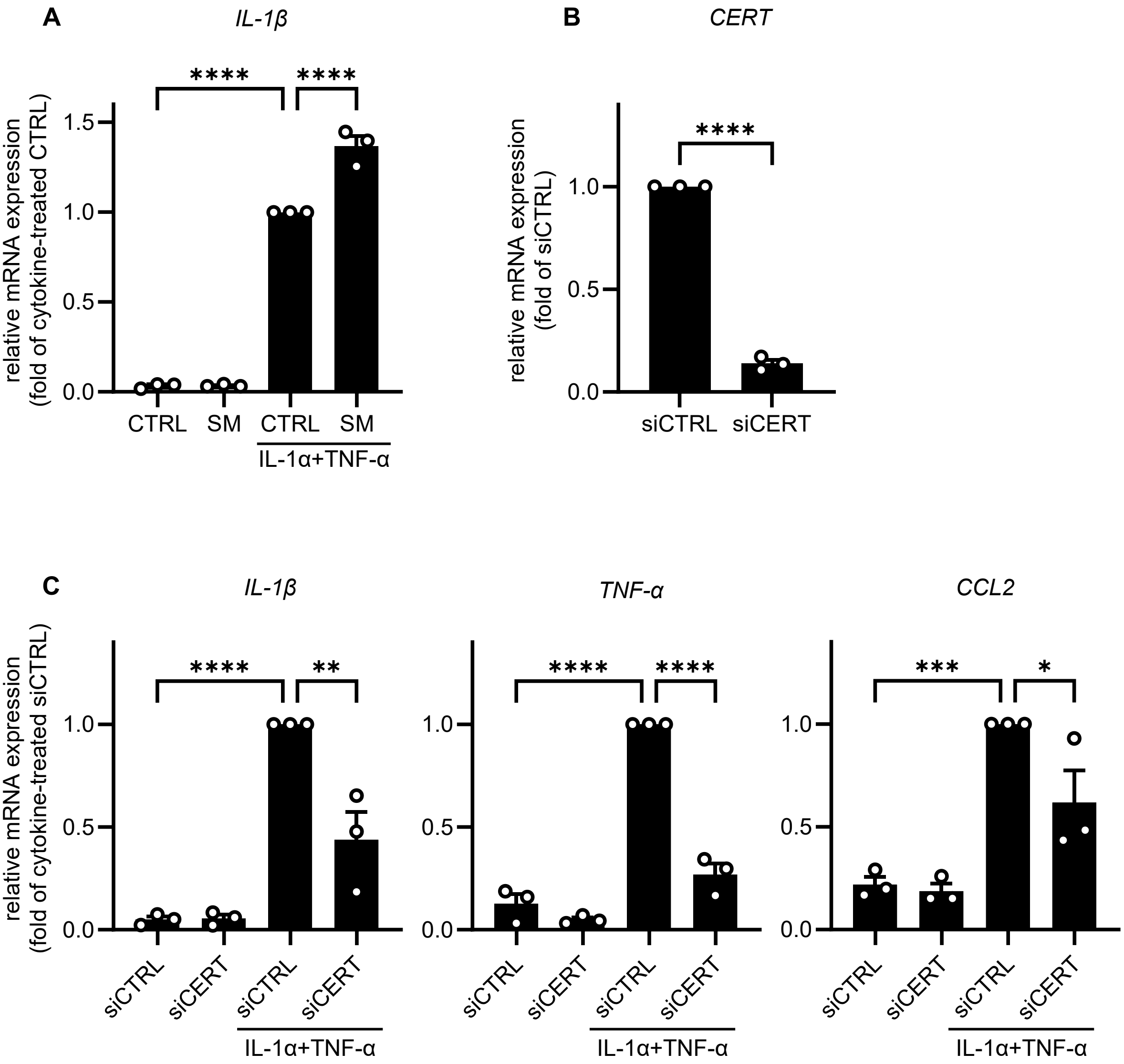

Supplementary Figure 10. The effects of changes in cellular SM levels on NF-κB signaling in human microglia-like cells

(A) HMC3 cells were treated with 1 μM SM for 48 h, and then co-treated with 1 μM SM, 3 ng/mL IL-1α, and 50 ng/mL TNF-α for 24 h. The mRNA expression levels of *IL-1β* were analyzed by qPCR (n=3). **(B)** HMC3 cells were transfected with control siRNA (siCTRL) or siRNA against CERT (siCERT) for 48 h. The knockdown efficiency was confirmed by qPCR (n=3). **(C)** CERT-knockdown cells were further incubated with 3 ng/ml IL-1α and 50 ng/ml TNF-α for 24 h. The mRNA expression levels of *IL-1β*, *TNF-α*, and *CCL2* were estimated by qPCR (n=3). All data are presented as mean ± SEM. A and C, Tukey's test. B, Student's t-test. * $P < 0.05$ ** $P < 0.01$ *** $P < 0.001$ **** $P < 0.0001$.

Supplementary Figure 11

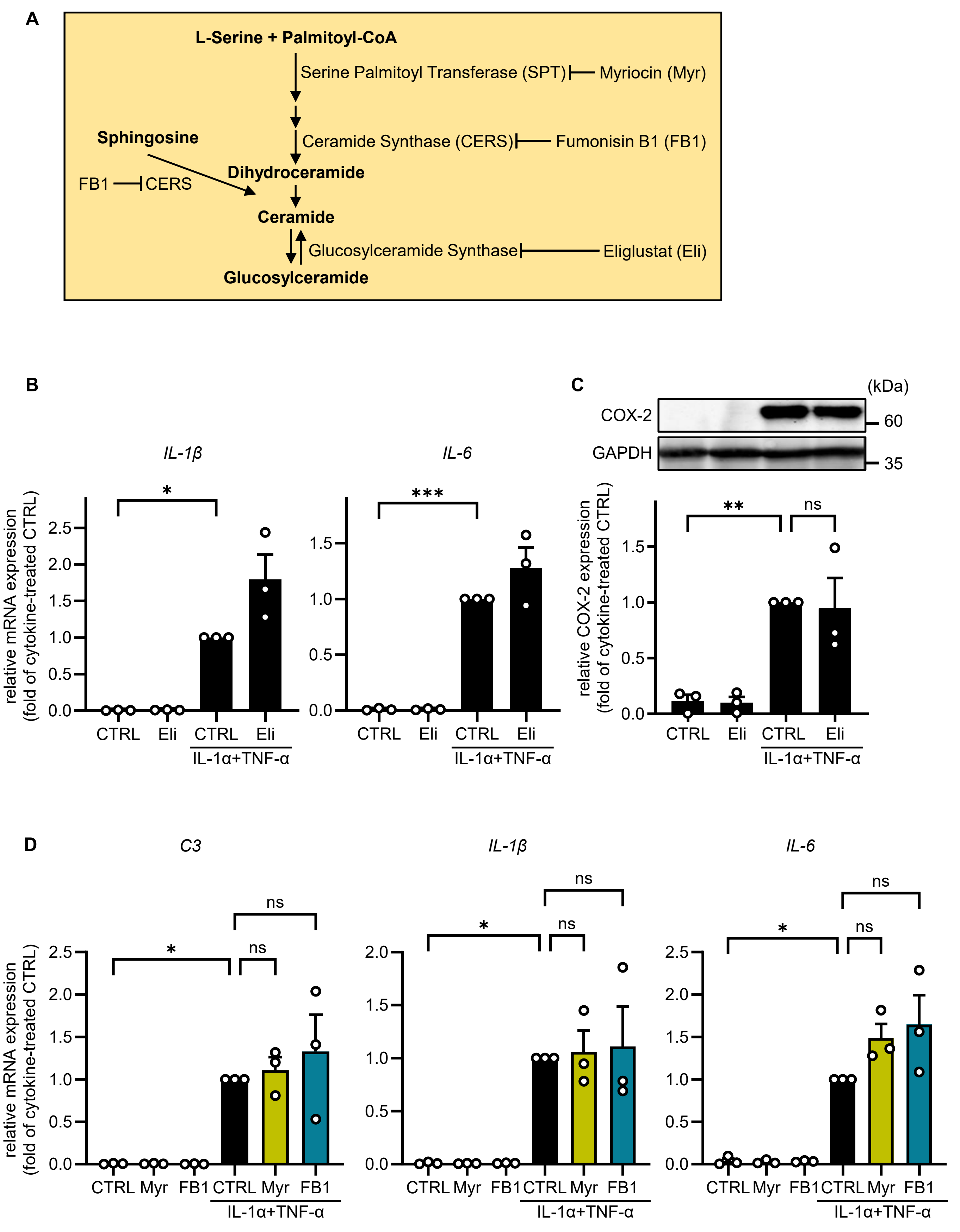

Supplementary Figure 11. The effects of sphingolipid metabolism inhibitors on astrocyte activity

(A) Sphingolipid metabolism and its inhibitors. **(B)** HASTR/ci35 were treated with 3 μM eliglustat (Eli) for 48 h in SFAM, and then the cells were further incubated with 3 μM eliglustat, 3 ng/ml IL-1α, and 50 ng/ml TNF-α for 24 h. The mRNA expression levels of *IL-1β* and *IL-6* were analyzed by qPCR (n=3). **(C)** The protein expression levels of COX-2 were assessed by western blot (n=3). **(D)** HASTR/ci35 cells were challenged with 1 μM myriocin (Myr) or 1 μM fumonisin B1 (FB1) for 48 h. The cells were then incubated with 1 μM myriocin or 1 μM fumonisin B1 with or without 3 ng/ml IL-1α and 50 ng/ml TNF-α for 24 h. The mRNA expression levels of the indicated genes were analyzed by qPCR (n=3). All data are presented as mean ± SEM. B, C, and D, Tukey's test. * $P < 0.05$ *** $P < 0.001$. ns, not significant.

Supplementary Figure 12

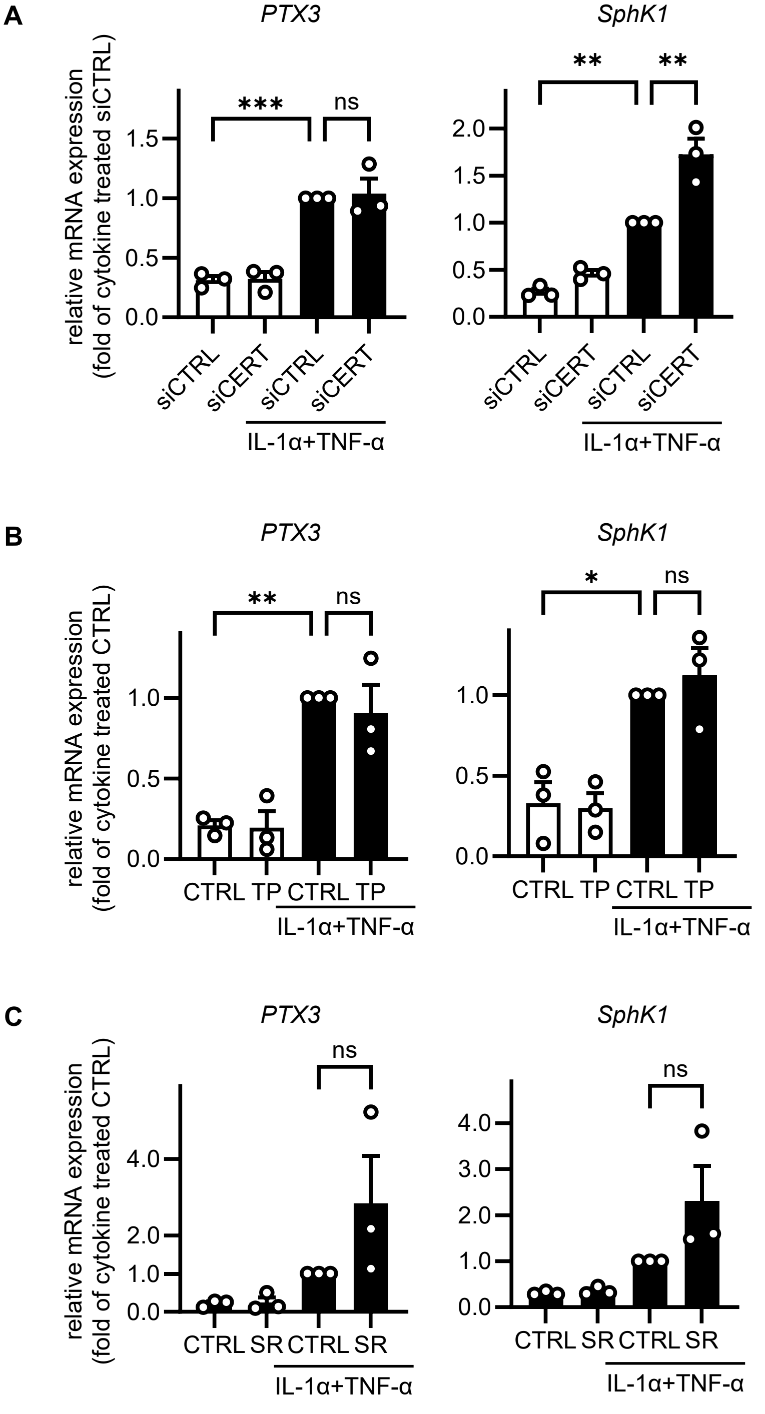

Supplementary Figure 12. CERT knockdown does not suppress neuroprotective astrocyte activation

(A) HASTR/ci35 cells were treated with control siRNA or CERT siRNA for 48 h. The cells were then incubated with 3 ng/ml IL-1α and 50 ng/ml TNF-α with SM for 24 h. The mRNA levels of *PTX3* and *SphK1* (neuroprotective astrocyte activation markers) were quantified by qPCR (n=3). **(B)** and **(C)** HASTR/ci35 cells were treated with 1 μM TPCA-1 (TP) or 1 μM SR11302 (SR) for 48 h in SFAM. The cells were then incubated with 3 ng/ml IL-1α and 50 ng/ml TNF-α for 24 h. The mRNA levels of *PTX3* and *SphK1* were quantified by qPCR (n=3). All data are presented as mean ± SEM. All data were analyzed by Tukey's test. * $P < 0.05$ ** $P < 0.01$ *** $P < 0.001$. ns, not significant.

Supplementary method

Cell culture of HMC3 cells

Human microglia-like cell line HMC3 cells were purchased from ATCC® (Cat# CRL-3304; Manassas, VA, USA) and routinely cultured at 37°C in an atmosphere of 5% CO_2/95% air in DMEM containing 10% FBS, 1% penicillin-streptomycin.
